# The N(^2^D) + CH_2_CHCN (Vinyl Cyanide) Reaction: A Combined Crossed Molecular Beam and Theoretical Study and Implications for the Atmosphere of Titan

**DOI:** 10.1021/acs.jpca.2c04263

**Published:** 2022-09-02

**Authors:** Gianmarco Vanuzzo, Demian Marchione, Luca Mancini, Pengxiao Liang, Giacomo Pannacci, Pedro Recio, Yuxin Tan, Marzio Rosi, Dimitrios Skouteris, Piergiorgio Casavecchia, Nadia Balucani

**Affiliations:** ∥ Dipartimento di Chimica, Biologia e Biotecnologie, Università degli Studi di Perugia, 06123 Perugia, Italy; ‡ Dipartimento di Ingegneria Civile e Ambientale, Università degli Studi di Perugia, 06125 Perugia, Italy; § Master-Tec srl, Via Sicilia 41, 06128 Perugia, Italy

## Abstract

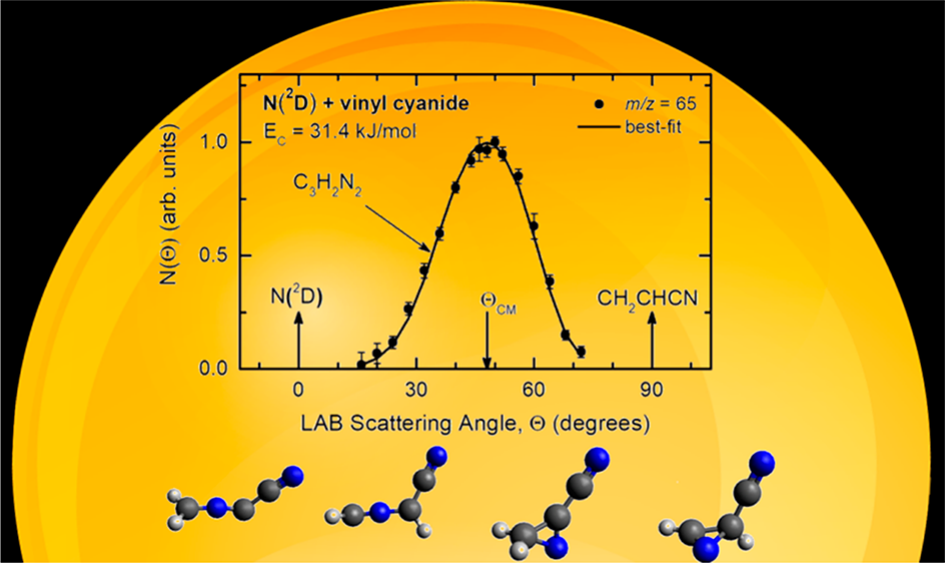

The reaction of electronically excited nitrogen atoms, N(^2^D), with vinyl cyanide, CH_2_CHCN, has been investigated under single-collision conditions by the crossed molecular beam (CMB) scattering method with mass spectrometric detection and time-of-flight (TOF) analysis at the collision energy, *E*_c_, of 31.4 kJ/mol. Synergistic electronic structure calculations of the doublet potential energy surface (PES) have been performed to assist in the interpretation of the experimental results and characterize the overall reaction micromechanism. Statistical (Rice–Ramsperger–Kassel–Marcus, RRKM) calculations of product branching fractions (BFs) on the theoretical PES have been carried out at different values of temperature, including the one corresponding to the temperature (175 K) of Titan’s stratosphere and at a total energy corresponding to the *E*_c_ of the CMB experiment. According to our theoretical calculations, the reaction is found to proceed via barrierless addition of N(^2^D) to the carbon–carbon double bond of CH_2_=CH–CN, followed by the formation of cyclic and linear intermediates that can undergo H, CN, and HCN elimination. In competition, the N(^2^D) addition to the CN group is also possible via a submerged barrier, leading ultimately to N_2_ + C_3_H_3_ formation, the most exothermic of all possible channels. Product angular and TOF distributions have been recorded for the H-displacement channels leading to the formation of a variety of possible C_3_H_2_N_2_ isomeric products. Experimentally, no evidence of CN, HCN, and N_2_ forming channels was observed. These findings were corroborated by the theory, which predicts a variety of competing product channels, following N(^2^D) addition to the double bond, with the main ones, at *E*_c_ = 31.4 kJ/mol, being six isomeric H forming channels: *c*-CH(N)CHCN + H (BF = 35.0%), *c*-CHNCHCN + H (BF = 28.1%), CH_2_NCCN + H (BF = 26.3%), *c*-CH_2_(N)CCN(cyano-azirine) + H (BF = 7.4%), *trans*-HNCCHCN + H (BF = 1.6%), and *cis*-HNCCHCN + H (BF = 1.3%), while C–C bond breaking channels leading to *c*-CH_2_(N)CH(2H-azirine) + CN and *c*-CH_2_(N)C + HCN are predicted to be negligible (0.02% and 0.2%, respectively). The highly exothermic N_2_ + CH_2_CCH (propargyl) channel is also predicted to be negligible because of the very high isomerization barrier from the initial addition intermediate to the precursor intermediate able to lead to products. The predicted product BFs are found to have, in general, a very weak energy dependence. The above cyclic and linear products containing an additional C–N bond could be potential precursors of more complex, N-rich organic molecules that contribute to the formation of the aerosols on Titan’s upper atmosphere. Overall, the results are expected to have a significant impact on the gas-phase chemistry of Titan’s atmosphere and should be properly included in the photochemical models.

## 1 Introduction

After the Pioneer 11, the two Voyager, and the Cassini–Huygens missions complemented by ground-based observations^1−4^ (including recent ones by the Atacama Large Millimeter/submillimeter Array, ALMA^5^), we have reached an unprecedented level of knowledge of the atmosphere of Titan, the largest moon of Saturn. The Cassini–Huygens mission confirmed that molecular nitrogen (N_2_) is by far the main component (∼94%) with methane accounting for a few percent.^6^ More complex organic molecules and the presence of nitriles in trace amounts have also been confirmed, and new species have been identified for the first time.^6,7^ Since the Voyager missions, Titan has been known to be covered by a thick orange haze,^7,8^ the origin of which has been a challenge for the scientific community. After the Cassini–Huygens mission, we now know that the first haze layer is at a much higher altitude than what was previously thought. Furthermore, the aerosol analyzer onboard Huygens indicated that the aerosols are nitrogen-rich organic macromolecules (with the C/N/H ratio being ca. 1:1:1).^8,9^ Another remarkable achievement of the Cassini–Huygens mission is the discovery of an unexpectedly rich ionosphere^10^ with large positive^11^ and negative^12^ ions with masses up to 8000 Da.

Since N_2_ has a very high chemical stability, the growth of N-containing molecules in planetary atmospheres (not only Titan but also Pluto) must be initiated by some form of active nitrogen, such as N atoms, the electronically excited *A*^3^Σ_u_^+^ of N_2_, and nitrogen ions (N^+^, N^2+^, N_2_^+^, and N_2_^+2^). These transient species can be easily formed starting from N_2_ in several processes involving energetic photons or particles, such as electron impact, extreme ultraviolet (EUV) photolysis, dissociative photoionization, and cosmic ray induced dissociation.^13−15^ All the processes mentioned above (plus the N_2_^+^ electron recombination) produce atomic nitrogen in the ground (^4^S_3/2_) and first electronically excited (^2^D_3/2,5/2_) states in similar amounts. Because of its spin-multiplicity, N(^4^S_3/2_) is relatively unreactive with closed-shell molecules while atomic nitrogen in the first electronically excited metastable state, ^2^D_3/2,5/2_, can react much more efficiently^13,16,17^ (energy content of 230.0 kJ/mol; radiative lifetimes of 6.1 × 10^4^ and 1.4 × 10^5^ s for ^2^D_3/2_ and ^2^D_5/2_, respectively^18^). It should be noted that the second excited metastable state of the N atom, the ^2^P state (energy content of about 345 kJ/mol; radiative lifetime of about 11 s), which can also be formed by the above energetic processes, is much less reactive than N(^2^D), and it mainly undergoes physical quenching in the conditions of the atmosphere of Titan.^13^ The reactions of N(^2^D) with the most abundant hydrocarbons have already been incorporated into photochemical models of Titan’s atmosphere.^19−23^ However, many key reactions remain unknown or not properly implemented in models, because of the lack of laboratory experiments and accurate theoretical calculations. For this reason, in our laboratory, we have started a systematic investigation of the reactions involving N(^2^D) and molecules that are known to be present in the atmosphere of Titan. More specifically, we have used the crossed molecular beam (CMB) technique with mass spectrometric (MS) detection and time-of-flight (TOF) analysis to identify the primary reaction product(s), their formation pathway(s) and, in the case of multichannel reactions, their branching fractions (BFs).^24^ Our experimental results have been accompanied by electronic structure calculations of the underlying potential energy surface (PES), and RRKM (Rice–Ramsperger–Kassel–Marcus) estimates of the product branching fractions (BFs) have been carried out under the conditions of our experiments and at the temperatures of relevance for Titan. The reactions N(^2^D) + CH_4_, C_2_H_2_, C_2_H_4_, C_2_H_6_, and C_3_H_4_ isomers (methylacetylene and allene (unpublished results))^25−31^ as well as N(^2^D) + small aromatic compounds (pyridine, benzene, and toluene)^32−36^ have been investigated. In a few cases, that is, for the N(^2^D) reactions with H_2_ and H_2_O, more sophisticated dynamical calculations have been performed and the detailed reaction mechanism elucidated.^37−40^

In all the reactions involving organic coreactants, new species holding a novel C–N bond were identified as major reaction products, thus implying that N(^2^D) reactions with hydrocarbons significantly contribute to the formation of the N-containing organic molecules. More recently, we have focused our attention to N(^2^D) reactions with nitriles. Nitriles, such as CH_3_CN, C_2_H_3_CN, C_2_H_5_CN, and HCCCN, have been observed in the atmosphere of Titan and reach a certain abundance at altitudes above 1000 km, a region where N(^2^D) is also formed by the high energy processes mentioned above. The first reaction we have looked at is the one involving cyanoacetylene, HCCCN.^41^ We have verified that the formation of dicyanocarbene, NCCCN, is the main reaction channel, at odds with respect to what is considered in most recent models^23^ where three possible product channels are instead assumed for the N(^2^D) + HCCCN reaction: (i) N_2_ + *c*-C_3_H, (ii) N_2_ + *l*-C_3_H, and (iii) C_2_N + HCN. Here, we extend the same combined experimental and theoretical approach to the reaction N(^2^D) + CH_2_CHCN (vinyl cyanide). Given the relevance of nitriles and of N-rich organic molecules in prebiotic chemistry, the work presented here is part of the Italian National Project of Astrobiology.^42^

The presence of vinyl cyanide (CH_2_CHCN, also known as cyanoethylene, acrylonitrile, or the IUPAC name 2-propenenitrile) in the atmosphere of Titan was first inferred by the detection of the cation CH_2_CHCNH^+^ using the Ion and Neutral Mass Spectrometer (INMS)^43−46^ aboard the Cassini orbiter. The definitive proof for the presence of the neutral CH_2_CHCN itself on Titan came from the detection by ALMA of its rotational lines in the frequency range of 230 to 232 GHz.^5^ A follow-up study using higher sensitivity data from the ALMA archive presented the very first spatially resolved map of the distribution of vinyl cyanide in Titan’s atmosphere.^47^ Among the other nitriles, vinyl cyanide received a great deal of attention because it is believed to be capable of forming membrane-like structures in nonpolar solvents, including methane which is the main constituent of Titan’s lakes.^48^ The molar fraction of vinyl cyanide is (3.4 ± 0.51) × 10^–7^ at 1050 km as inferred using the INMS in closed-source neutral (CSN) mode and by fitting the INMS CSN signal using cracking patterns of multiple species.^45^ Notably, a higher molar fraction of about 10^–5^ at 1100 km was reported by Vuitton et al.^44^ with a value that is closer to the predicted abundances of recent models.^5,19^ This second estimate has also been confirmed by the ALMA data.

In the photochemical models of Titan, the main loss mechanisms of vinyl cyanide are (1) proton transfer from HCNH^+^ and C_2_H_5_^+^ followed by electron recombination of CH_2_CHCNH^+^,^49,50^ (2) photodissociation, mainly leading to cyanoacetylene (HCCCN), (3) condensation, (4) downward transport, and (5) H atom addition. The very same models show molar abundances of N(^2^D) as a function of the altitude (Figure 31 of ref (19)) with a value of ∼10^–6^ at similar altitudes where vinyl cyanide is most abundant. Therefore, although most of N(^2^D) will react with more abundant and simpler hydrocarbons, CH_2_CHCN will be consumed by the reaction with N(^2^D) as well. For this reason, the title reaction has been considered in the most recent photochemical models (such as those described in refs (19 and 23)). Since there are no data available for this system, the reaction has been introduced with estimated parameters. Loison et al.^23^ were the first to consider the N(^2^D) + CH_2_CHCN reaction. By analogy with the reaction N(^2^D) + C_2_H_4_, they have introduced an H-displacement channel leading to HNCCHCN + H with an estimated rate coefficient of 2.3 × 10^–10^ exp(−503/*T*) cm^3^ molec^–1^ s^–1^. In addition, they have included a reaction channel leading to N_2_ + C_3_H_3_ with an estimated rate coefficient of 4 × 10^–11^ cm^3^ molec^–1^ s^–1^ by analogy with the reaction N(^2^D) + HCN. In a more recent model, Vuitton et al.,^19^ instead, included the title reaction as N(^2^D) + C_3_H_3_N → H + C_3_H_2_N_2_, making no distinction between the possible isomeric products having the same empirical formula C_3_H_2_N_2_. Also in this case, an estimated rate coefficient of 2.3 × 10^–10^ exp(−503/*T*) cm^3^ molec^–1^ s^–1^ was employed. In both models, it is not clear which destiny HNCCHCN or C_3_H_2_N_2_ has (see the last section for further comments).

A final remark concerns the possible role of the title reaction in the nitrogen chemistry of the interstellar medium, which is still little understood. The forbidden emission doublet lines centered at 519.79 and 520.03 nm originating from metastable N(^2^D_3/2,5/2_) have been detected in a plethora of strongly photon-irradiated environments including the Orion Nebula (M42), low-ionization H II regions (M43), planetary nebulae (e.g., the Ring Nebula), supernova remnants (e.g., the Crab Nebula), and Herbig-Haro objects.^58−61^ At the same time, vinyl cyanide was found in Sagittarius B2,^51^ in the hot molecular core Sgr B2(N)^52,53^ in Orion-KL,^54^ in the TMC-1 dark cloud,^55^ in the L1544 proto-typical prestellar core,^56^ and the circumstellar envelope of the C-rich star IRC + 10216,^57^ suggesting that it is a common molecule in interstellar objects of various kinds. The title reaction, therefore, could also occur in specific regions of the interstellar medium.

In general, we can say that very little is known on the reactions of vinyl cyanide with atomic and radical species.^62,63^ The global rate coefficient is known for a few cases (H,^64^ Cl,^65^ CN,^66^ and OH^67,68^) while the product branching fractions (BFs) are mostly undetermined. For this reason, we have started a systematic investigation of the reactions involving vinyl cyanide and common atomic/diatomic radicals. The results concerning the reaction CN + CH_2_CHCN have just been published^63^ while other studies are still under way.

In this work, we report on a combined experimental and theoretical investigation of the reaction N(^2^D) + CH_2_CHCN. More specifically, we have employed the CMB technique to explore the nature of the primary products and their BFs. In addition, we have performed dedicated electronic structure calculations of the underlying PES and RRKM (Rice–Ramsperger–Kassel–Marcus) estimates of the product BFs to assist in the interpretation of the scattering data and to determine the reaction mechanism.

The paper is structured as follows. In Section 2, we describe the experimental and theoretical methods at the basis of our work. The presentation of the experimental and theoretical results will follow in Sections 3 and 4, respectively. The experimental and theoretical results are then discussed in Section 5, while the implications for the atmosphere of Titan will be commented on in Section 6. Finally, the conclusions will summarize the key points of the present study.

## 2 Experimental and Theoretical Methods

### 2.1 Experimental Method

The experiments were carried out using the “universal” CMB apparatus, which has been described in detail in refs (24 and 69−71). Briefly, two continuous supersonic beams of the reactants are crossed at the fixed angle of 90° under single collision conditions in a large scattering chamber kept at 7 × 10^–7^ hPa in operating conditions. The apparatus is characterized by a detector that can be rotated in the plane of the reactants, around the ideal axis passing though the collision center, where the laboratory angle Θ = 0° corresponds to the atomic nitrogen beam direction. The detector is a tunable electron-impact ionizer followed by a quadrupole mass filter and an ion counting Daly type system^72^ located in a triply differentially pumped UHV chamber (10^–11^ hPa). Atomic nitrogen was produced by a radio frequency (RF) discharge supersonic beam source, discharging 90 hPa of a 2.5% N_2_/He gas mixture through a 0.48 mm, water cooled quartz nozzle at 250 W of RF power,^73,74^ followed by a 0.8 mm diameter boron nitride skimmer. In these conditions, a high molecular dissociation (about 60%) is ensured.^75^ In particular, atomic nitrogen was produced in a distribution of electronic states, which was characterized in our laboratory by Stern-Gerlach analysis:^74^ 72% of N atoms are in the ^4^S ground state, while 21% and 7% are in the metastable excited ^2^D and ^2^P states, respectively. The resulting beam velocity and speed ratio were 2283 m/s and 5.4, respectively. In our previous experiments on the reactions of N atoms with saturated and unsaturared hydrocarbons,^25−31^ we verified that the presence of N(^4^S) and N(^2^P) in the nitrogen beam did not affect the experimental outcomes, because the rate coefficients of the N(^4^S) and N(^2^P) reactions with saturated and unsaturated hydrocarbons are much smaller than those of N(^2^D).^13,16,17^ In addition, the extra energy carried by the ^2^P state was never necessary to fit the experimental data. Analogous to the case of N(^2^D) + C_2_H_4_, we expect that the reactions involving N(^4^S) and N(^2^P) + vinyl cyanide do not contribute to the reactive signal. Indeed, the present experimental results are perfectly in line with the exclusive contribution of the N(^2^D) reaction.

The supersonic beam of vinyl cyanide was generated by expanding 105 hPa of neat gas (Merck 99.9% purity) through a 0.1 mm diameter stainless-steel (S.S.) nozzle, followed by a 0.8 mm S.S. skimmer. Liquid vinyl cyanide was kept in a reservoir immersed in a water/ethylene glycol bath (whose temperature was kept at about 293 K) to avoid temperature and then vapor pressure fluctuations. The peak velocity of the vinyl cyanide molecular beam was measured to be 663 m/s with a speed ratio of 3.8. In these conditions, the collision energy (*E*_c_) was 31.4 kJ/mol and the center-of-mass (CM) angle, Θ_CM_, of the system was 47.7°. The defining element, positioned after each skimmer, determined an angular divergence of 2.4° and 3.8° for the atomic nitrogen and vinyl cyanide beam, respectively.

Product angular distributions in the laboratory (LAB) coordinate system, N(Θ), were measured by modulating at 160 Hz the vinyl cyanide beam for background subtraction. Product velocity distributions, N(Θ, *t*), were measured by a pseudorandom chopping disk and the cross-correlation method at 6 μs/channel. All measurements were performed using hard (70 eV) electron ionization. Quantitative information is obtained by moving from the LAB coordinate system to the center-of-mass (CM) one and analyzing the product angular, *T*(θ), and translational energy, *P*(*E*_T_^′^), distributions into which the CM product flux can be factorized (the best-fit CM functions are actually derived by a forward convolution fit of the product LAB angular and TOF distributions).^24,69^

### 2.2 Theoretical Methods

**Figure 1:**
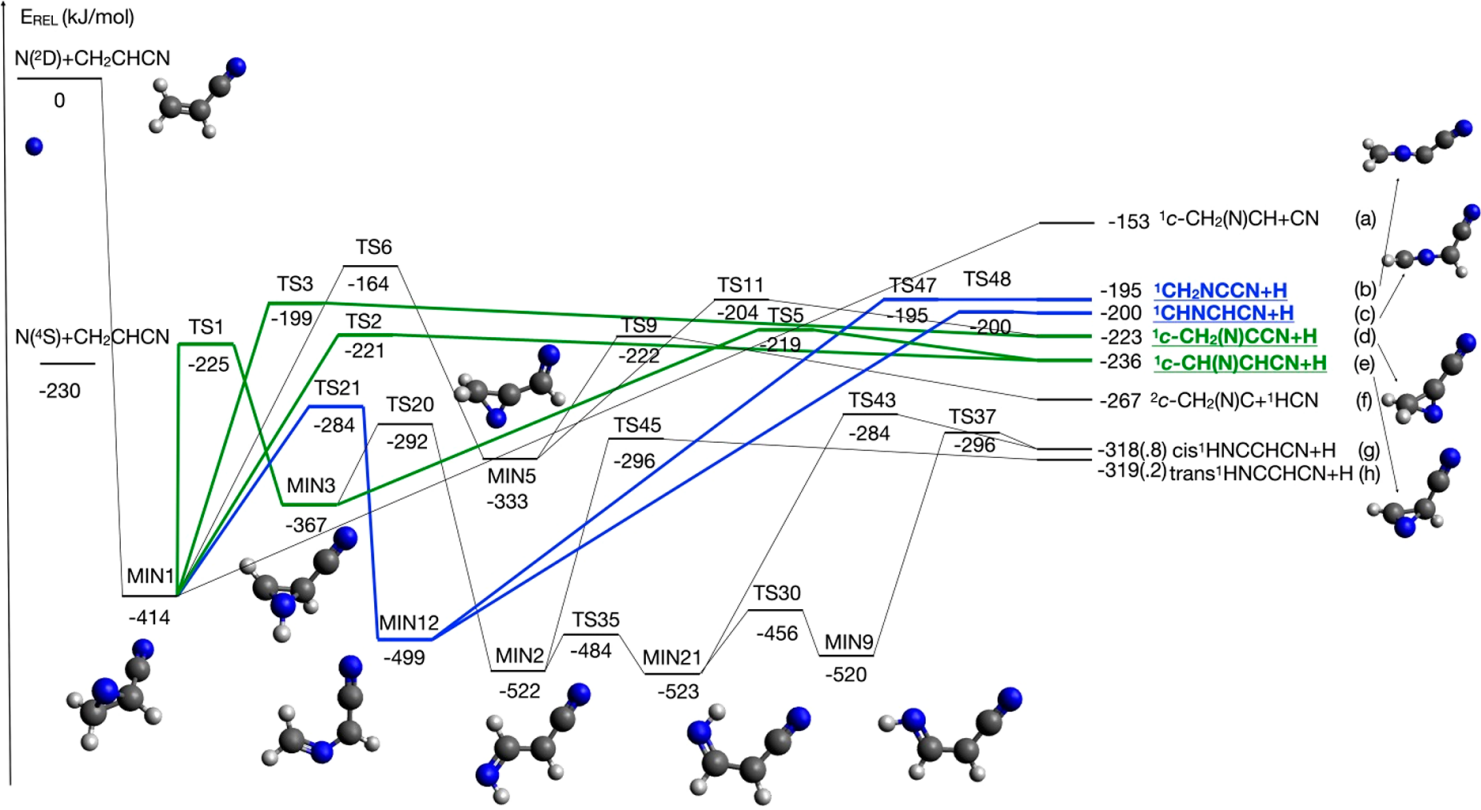
Schematic representation of the potential energy surface for the reaction N(^2^D) + CH_2_CHCN with energies evaluated at the CCSD(T)/aug-cc-pVTZ level of theory (see text), considering only N(^2^D) addition to the double bond. The structure of the heavier coproduct from the four main channels, all accompanied by the H coproduct, is shown as well as the structure of the main intermediates. Thick, color coded (green and blue) solid lines indicate the four pathways leading to the underlined main products according to our RRKM estimates (see text).

**Figure 2:**
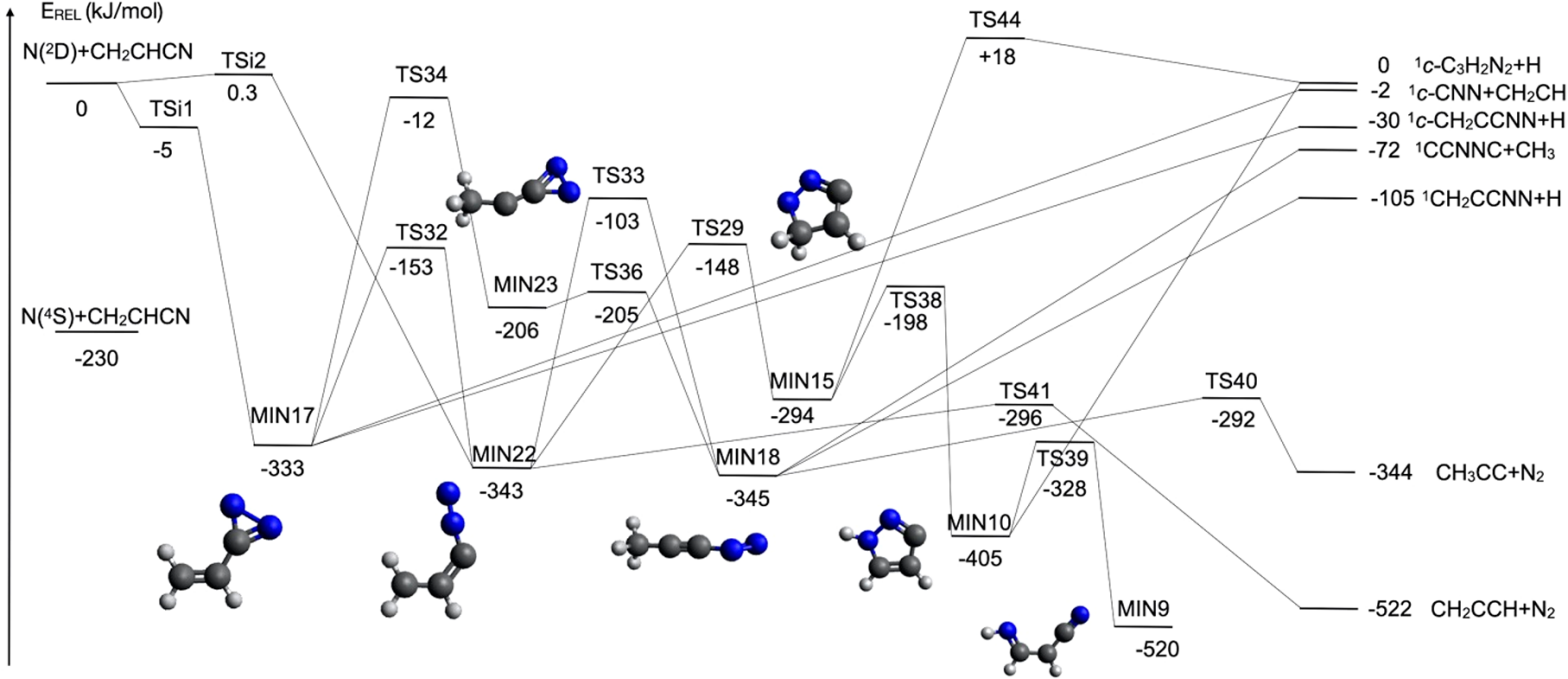
Schematic representation of the potential energy surface for the reaction N(^2^D) + CH_2_CHCN with energies evaluated at the CCSD(T)/aug-cc-pVTZ level of theory (see text). In this figure, we have considered the attack of the N(^2^D) atom to the triple bond of CN and to the lone pair of the nitrogen atom.

The doublet PES for the reaction N(^2^D) + CH_2_CHCN was investigated by adopting a computational strategy previously used for the characterization of other reactions.^25−29,76,77^ Density functional theory calculations with the hybrid functional B3LYP^78,79^ in conjunction with the correlation consistent valence polarized set aug-cc-pVTZ^80−82^ were performed to locate the lowest stationary points. The same level of theory was utilized to compute the harmonic vibrational frequencies to check the nature of the stationary points: that is, the minimum if all the frequencies are real; saddle point if there is one and only one, imaginary frequency. The assignment of the identified stationary points was performed through intrinsic reaction coordinate (IRC) calculations.^83,84^ The energy of all the identified stationary points was calculated using the more accurate coupled cluster theory, including single and double excitations as well as a perturbative estimate of connected triples CCSD(T)^85−87^ with the same basis set aug-cc-pVTZ. Both the B3LYP and the CCSD(T) energies were corrected to 0 K by adding the zero-point energy correction computed using the scaled harmonic vibrational frequencies evaluated at the B3LYP/aug-cc-pVTZ level. Since the accuracy of our best computed values should not be better than the “chemical accuracy” of 1 kcal/mol, we rounded all the reported energies to 1 kJ/mol. All calculations were performed using the Gaussian 09 software,^88^ while the visualization of the structures and the analysis of the vibrational frequencies were performed using AVOGADRO.^89,90^ The energy of N(^2^D) was evaluated by adding the experimental separation^91^ N(^4^S)–N(^2^D) of 230.0 kJ/mol to the energy of N(^4^S) at all levels of the calculation. Schematic diagrams of the resulting PES are depicted in Figures 1 and 2. Figure 1 reports those intermediates and transition states up to the product formation that are formed starting with the N(^2^D) addition to the olefinic π bond. Figure 2 reports intermediates and transition states up to the product formation when the attack of the N(^2^D) atom is directed toward the triple bond or the lone pair of the nitrogen atom of the nitrile group.

### 2.3 RRKM Calculations

RRKM calculations for the N(^2^D) + CH_2_CHCN reaction were performed using a code implemented in our group for this purpose.^29,30,92^ The microcanonical rate constant *k*(*E*) for a specific reaction at a specific total energy is given by the expression  where *N*(*E*) represents the sum of states at the transition state at energy *E*, ρ(*E*) is the density of states of the reactant, and *h* is Planck’s constant. *N*(*E*) is obtained by integrating the relevant density of states up to energy *E*, and the rigid rotor/harmonic oscillator model is assumed. Since many reaction channels are H-displacement processes, tunneling (as well as quantum reflection) was included in the RRKM calculations using the corresponding imaginary frequency of the transition state and calculating the tunneling probability for the corresponding Eckart barrier.

For the cases in which we were not able to locate a clear transition state in the exit channel, the corresponding microcanonical rate constant was obtained through a variational approach: *k*(*E*) was evaluated at various points along the reaction coordinate, and the point that minimizes the rate constant was chosen in accordance with the variational theory.^93^ For dissociation steps in which, due to difficulties in the electronic structure calculations, no intermediates points are available, the products at infinite separation were taken into account as a possible “transition state”.

After the calculation of all microcanonical rate constants, a Markov (stochastic) matrix was set up for all intermediates and final channels to derive the product branching fractions for the overall reaction. *k*(*E*) is subsequently Boltzmann averaged for each temperature of interest to yield *k*(*T*).

## 3 Experimental Results

According to the present theoretical calculations, the N(^2^D) + CH_2_CHCN reaction exhibits numerous, energetically open channels (see Figures 1 and 2) with the most exothermic ones being the following (the reported enthalpies of reaction are those calculated in the present work at the CCSD(T) level; see section 2.2):N(^2^D) + CH_2_CHCN →
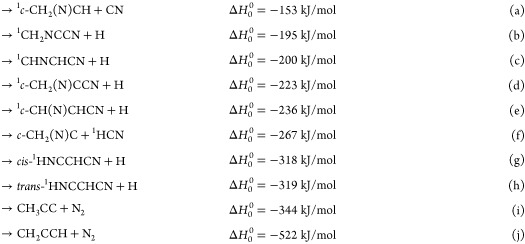


Experimentally, reactive scattering signal was detected only at the mass-to-charge ratio, *m*/*z*, of 66, 65, and 64, corresponding to the ions C_3_H_2_N_2_^+^ (associated with the parent ion of the products with gross formula C_3_H_2_N_2_), C_3_HN_2_^+^, and C_3_N_2_^+^, respectively. The signal relative intensities were ca. 0.02, 1.0, and 0.02. The scattering distributions recorded at the three *m*/*z*’s were superimposable, thus implying that H_2_-elimination channels are not present and that the reactive signal recorded in this range of masses is associated with one or more H-displacement channels. Because of the much better signal-to-noise ratio (S/N), all final measurements were carried out at *m*/*z* = 65. No reactive signal was detected at *m*/*z* = 39 and 38, which are parent and (−1) daughter ion, respectively, of the C_3_H_3_ (propargyl) or its isomer CH_3_CC, which can be produced in the two most exothermic channels (i) and (j), leading to N_2_ formation. Also, the formation of C_2_NH_3_ and C_2_NH_2_ products from the CN and HCN forming channels (a) and (f) was investigated by attempting detection at *m*/*z* = 41 and 40. Again, no reactive signal was observed. Within the sensitivity of our experimental method, the above puts the BFs of the N_2_, CN, and HCN forming channels to less than 5%.

**Figure 3:**
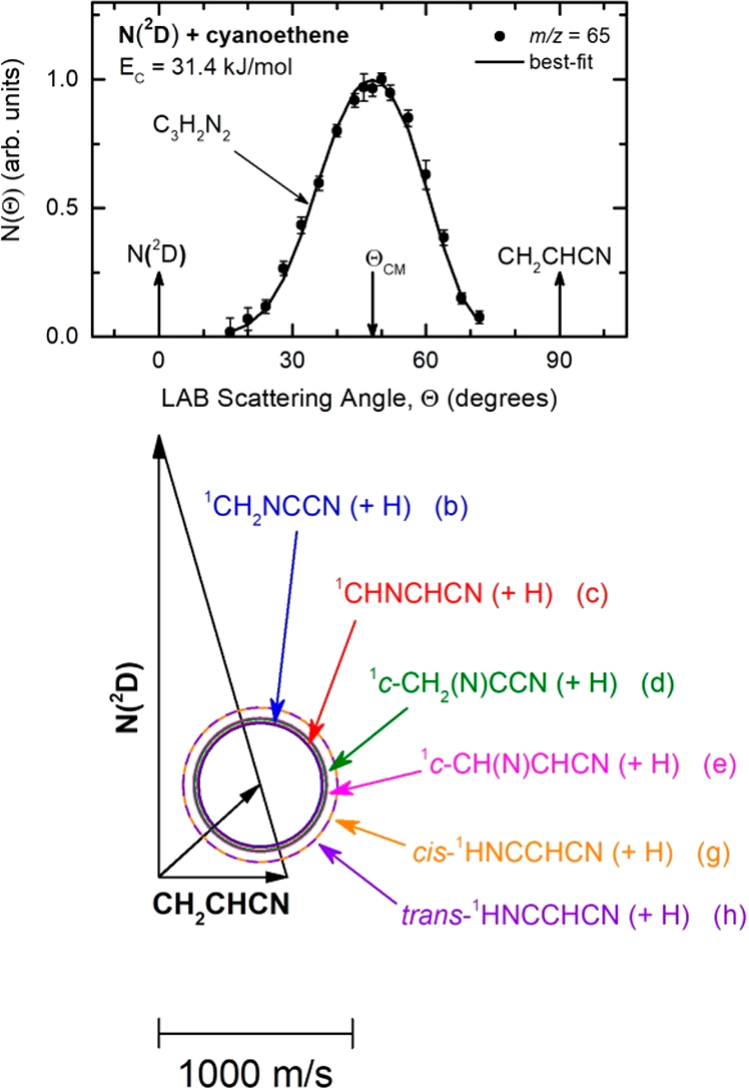
(Top): LAB angular distribution of the C_3_H_2_N_2_ product detected at *m*/*z* = 65 (C_3_HN_2_^+^) for the N(^2^D) + vinyl cyanide reaction at *E*_c_ = 31.4 kJ/mol. The solid black curve represents the calculated distribution when using the best-fit CM functions shown in Figure 5. (Bottom): Velocity vector (Newton) diagram of the experiment. The radius of each circle represents the maximum velocity that the indicated product can attain in the center-of-mass (CM) reference frame if all available energy is channeled into product recoil energy (see text).

**Figure 4:**
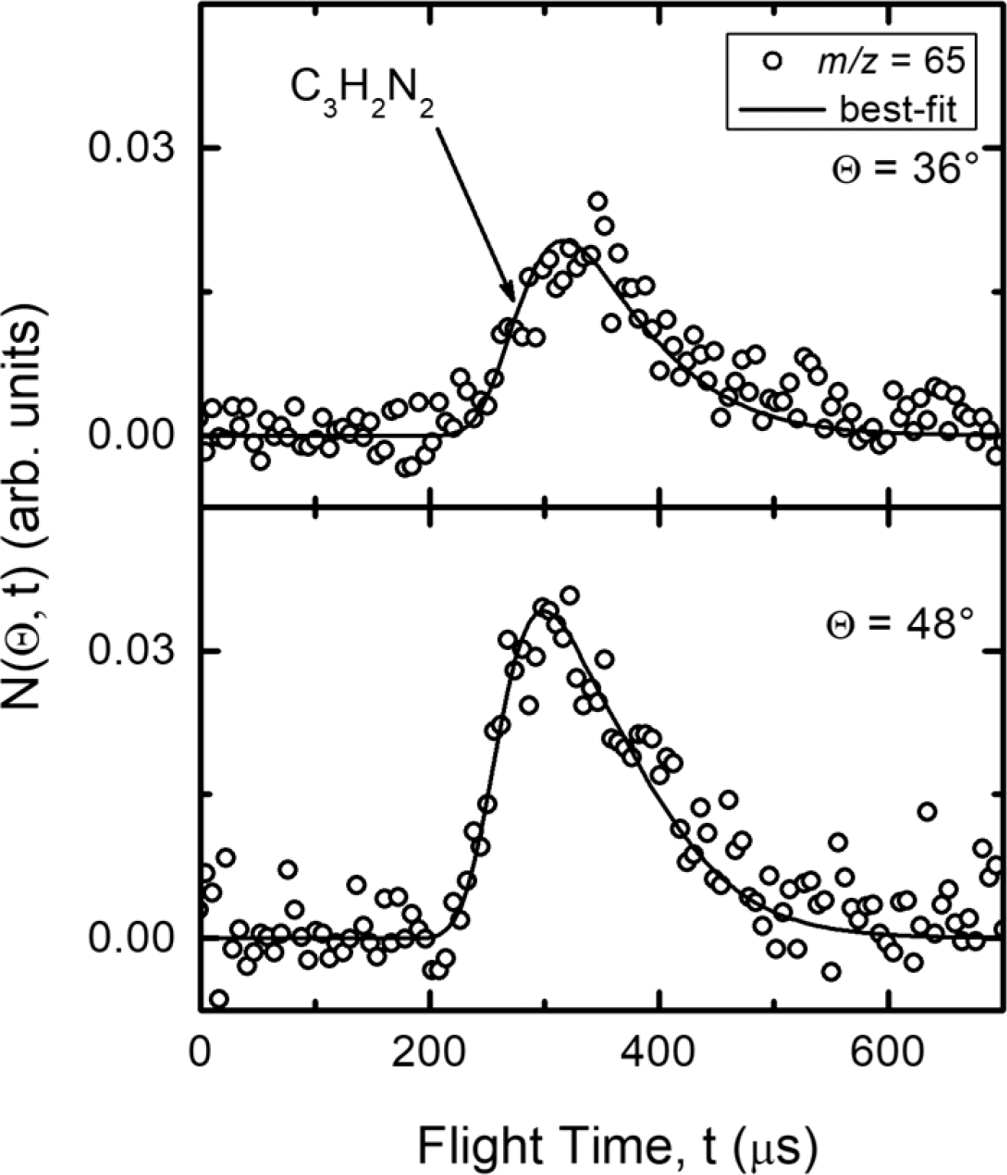
Time-of-flight distributions at *m*/*z* = 65 at the indicated LAB angles for the N(^2^D) + vinyl cyanide reaction. Empty circles: experimental data. Solid line: simulated TOF spectra when using the best-fit CM functions depicted in Figure 5.

**Figure 5:**
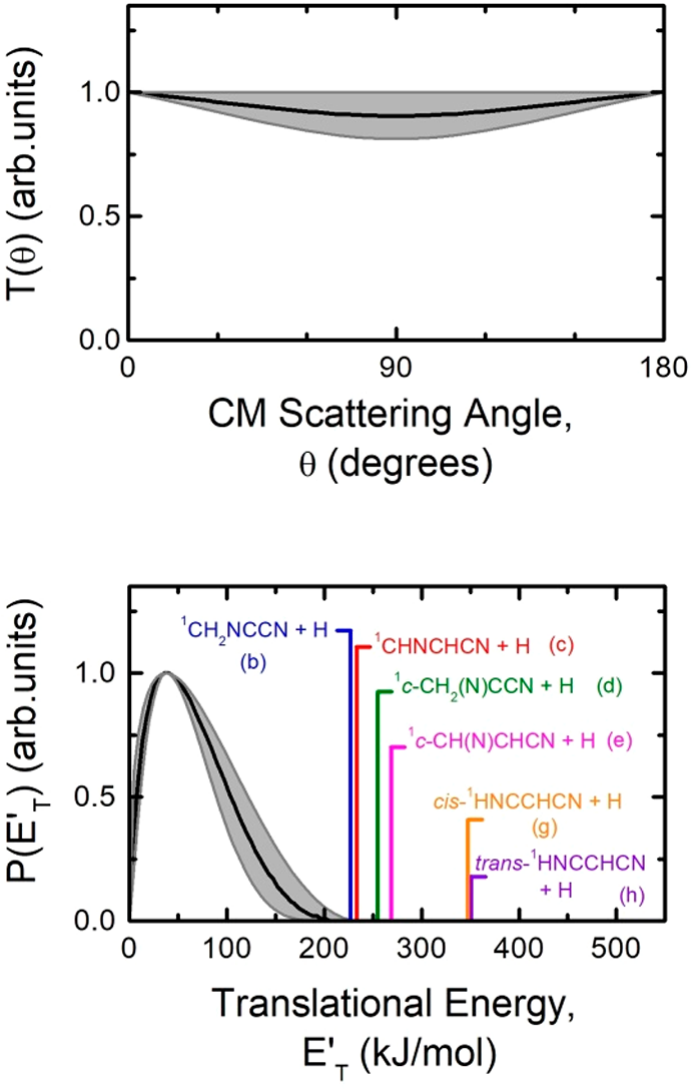
Best-fit CM product angular, *T*(θ), (top) and translational energy, *P*(*E*_T_^′^), (bottom) distributions for the N(^2^D) + C_2_H_3_CN reaction. The shaded areas represent the error bars determined for the best-fit CM functions. The vertical lines in the graph of *P*(*E*_T_^′^) indicate the total energy (*E*_tot_ = *E*_c_ – Δ*H*_0_^0^) of the six different, most exothermic H-displacement isomeric channels (b), (c), (d), (e), (g), and (h), in which the heavy coproduct of the H atom has general formula C_3_H_2_N_2_.

The velocity vector (so-called “Newton”) diagram for the title reaction is depicted in Figure 3 (bottom), where the superimposed circles correspond to the maximum CM speed that each (indicated) product among the six most important H-displacement channels (b), (c), (d), (e), (g), and (h) can attain by assuming that all the total available energy (given by *E*_C_ – Δ*H*_0_^0^) is channelled into product translational energy. The *m*/*z* = 65 LAB angular distribution is reported in Figure 3 (top). The filled circles indicate the intensity averaged over five different scans (with a counting time of 50 s at each angle) while the error bars represent the ±1 standard deviation. The experimental angular distribution is bell-shaped, centered around 48° (Θ_CM_ = 47.7°). Product TOF distributions at *m*/*z* = 65 were recorded at two different angles (Θ = 36° and Θ = 48°) and are shown in Figure 4. The single peak (peak position around 310 μs) without a significant structure indicates that it is not possible to disentangle the possible H-displacement isomeric products(s) (see Figure 3 (bottom)). The solid curves superimposed over the experimental data of Figures 3 and 4 are the simulated distributions when using only one set of CM distributions. The best-fit CM angular, *T*(θ), and translational energy, *P*(*E*_T_^′^), distributions are displayed in Figure 5.

The characteristics of the best-fit *P*(*E*_T_^′^) are compatible with all six isomeric H-displacement products associated with the channels (b), (c), (d), (e), (g), and (h) (see Figure 5 (bottom)). The product translational energy distribution peaks around 37–38 kJ/mol and is characterized by a cutoff at a total energy of 200 ± 25 kJ/mol. The best-fit CM angular distribution is distributed over the entire 0–180 °CM angular range and is backward–forward symmetric (Figure 5 (top)). This indicates that the C_3_H_2_N_2_ product(s) is (are) formed via a “long-lived complex” mechanism (i.e., a complex living at least 5–6 rotational periods according to the “osculating complex” model for chemical reactions).^94−96^ Incidentally, this fully justifies the adoption of the RRKM statistical method for the estimates of the product BFs (see Section 4.2).

H-displacement can occur through different pathways giving different isomeric products. Since the differences in exothermicity are small and the reaction mechanism is expected to be similar for the various H-displacement channels, we have not been able to disentangle the different contributions.

The experimental average product translational energy, defined as , is 62 kJ/mol, and the fraction of the total available energy, *E*_TOT_, channelled in product translational energy, , is 0.27 when using the theoretical value of the least exothermic H-displacement channel (b) for *E*_TOT_ (*E*_TOT_ = *E*_C_ – Δ*H*_0_^0^), leading to ^1^CH_2_NCCN + H (Δ*H*_0_^0^ = −195 kJ/mol) or even smaller than 0.27 if we refer to the more exothermic H channels. It follows that the C_3_H_2_N_2_ product(s) is (are) formed with a significant fraction of ro-vibrational excitation.

## 4 Theoretical Results

### 4.1 Electronic Structure Calculations

#### 4.1.1 Reactive Pathways Starting with the N(^2^D) Addition to the Olefinic π Bond

**Figure 6:**
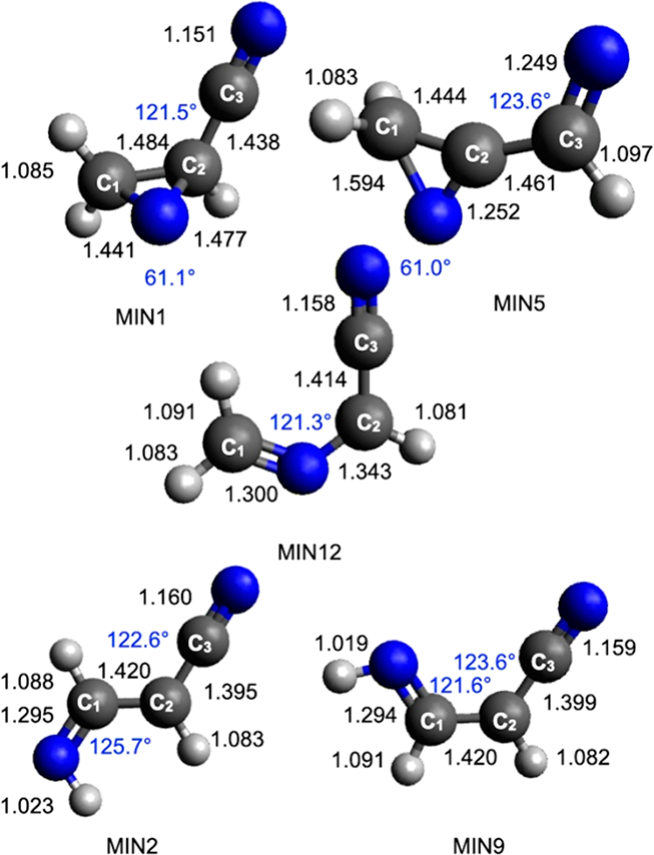
B3LYP optimized geometries (distances in black in Å, angles in blue in degrees) of the minima identified along the PES for the reaction N(^2^D) + vinyl cyanide CH_2_CHCN.

**Figure 7:**
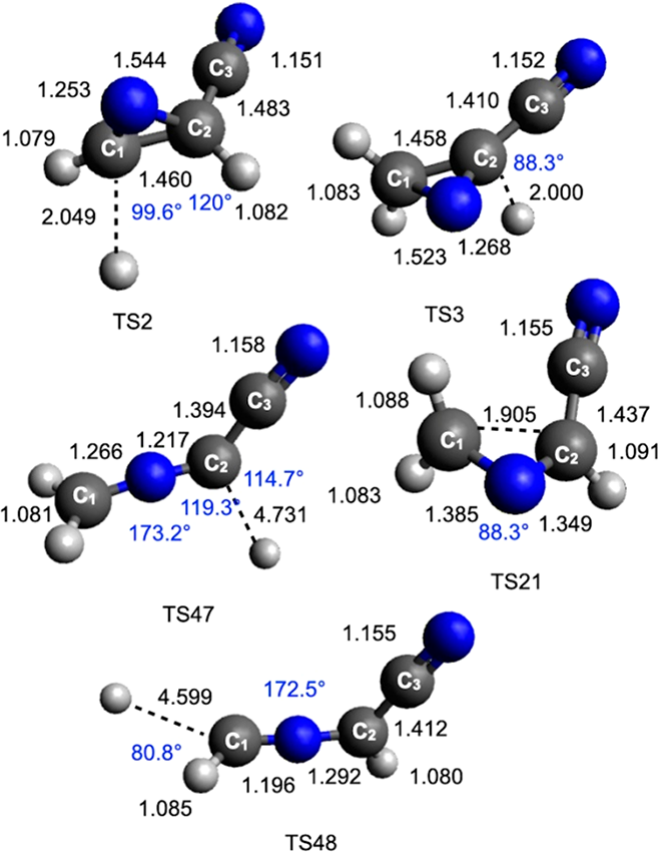
B3LYP optimized geometries (distances in black in Å, angles in blue in degrees) of the main transition states identified along the PES for the reaction N(^2^D) + CH_2_CHCN.

**Figure 8:**
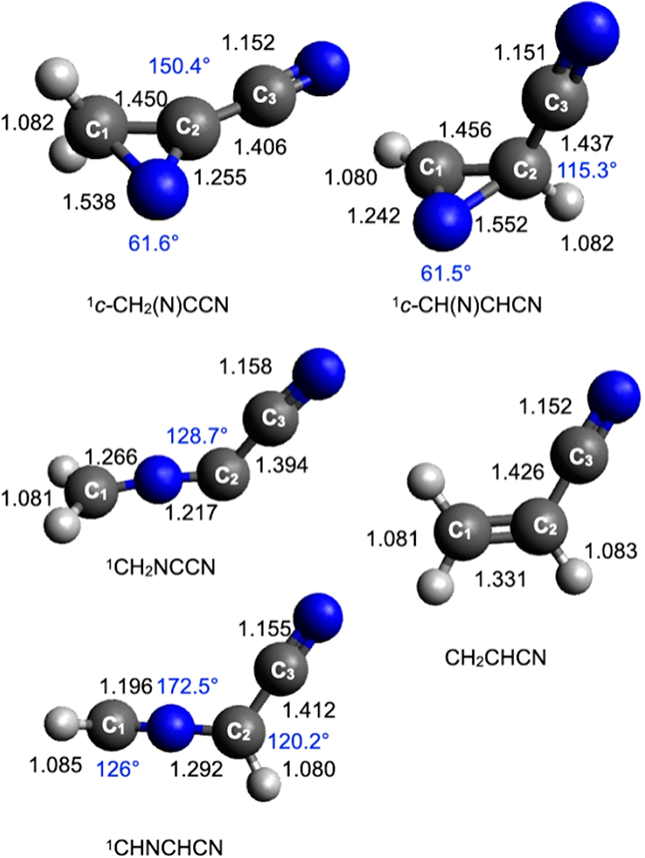
B3LYP optimized geometries (distances in black in Å, angles in blue in degrees) of vinyl cyanide and of the four main molecular products identified along the PES for the reaction N(^2^D) + CH_2_CHCN.

As we are going to see, this is the most efficient reactive approach and is similar to what we have already seen in the case of the N(^2^D) reaction with ethylene.^27,29^ However, because of the symmetry loss, the global PES is even more complex and features 23 different minima, linked by 25 possible transition states. A total number of 47 possible sets of products formed in 47 reaction channels can be formed with energy paths entirely located below the reactant energy asymptote. For the sake of clarity, in Figure 1, we have reported a simplified version of the PES, considering only the reactive channels leading to appreciable values of product BFs as a result of our RRKM calculations (see Section 4.2). A global overview including all the possible isomerization processes together with all the energetically accessible products is reported in the Supporting Information. The geometry of the most relevant stationary points (including intermediates, transition states, reactants, and products) is shown in Figures 6, 7, and 8, while Table 1 reports the reaction enthalpies and barrier heights computed at 0 K (with the inclusion of the zero-point energy correction) for the most important channels.

**Table 1:** Reaction Enthalpy, Δ*H*_0_^0^ (kJ/mol), and Barrier Height (kJ/mol, 0 K) at the CCSD(T)/aug-cc-pVTZ//B3LYP/aug-cc-pVTZ Level of Theory for all the Isomerization and Dissociation Steps of the N(^2^D) + CH_2_CHCN PES

process	Δ*H*_0_^0^ (kJ/mol)	barrier height (kJ/mol)
N(^2^D) + CH_2_CHCN → MIN1	–414	
MIN1 → MIN3	47	189
MIN1 → MIN5	81	250
MIN1 → MIN12	–85	130
MIN1 → ^1^*c*-CH_2_(N)CCN + H	191	215
MIN1 → ^1^*c*-CH(N)CHCN + H	178	193
MIN1 → ^1^*c*-CH_2_(N)CH + CN	261	none
MIN3 → ^1^*c*-CH(N)CHCN + H	131	148
MIN3 → MIN2	–155	75
MIN2 → *trans*-^1^HNCCHCN + H	203	226
MIN2 → MIN21	–1	38
MIN21 → *cis*-^1^HNCCHCN + H	204	239
MIN21 → MIN9	3	67
MIN9 → *cis*-^1^NHCCHCN + H	201	224
MIN5 → ^1^*c*-CH_2_(N)C + HCN	66	111
MIN5 → ^1^*c*-CH_2_(N)CCN + H	110	129
MIN12 → ^1^CH_2_NCCN + H	304	304
MIN12 → ^1^CHNCHCN + H	299	299

As can be seen in Figure 1, the addition of N(^2^D) to the double bond of CH_2_CHCN is barrierless and leads to the formation of the cyclic intermediate MIN1 (located at −414 kJ/mol with respect to the reactant energy asymptote). Once formed, MIN1 can directly undergo a C–H bond fission. Two different combinations of products can be formed, depending on which C–H bond fission occurs, namely, that involving the terminal C_1_ carbon atom (see Figure 6 for numeration of the carbon atoms) or that involving the central C_2_ carbon atom. In the first case, the cyclic coproduct ^1^*c*-CH(N)CHCN is formed (channel (e)) through an exit barrier (TS2) located at +193 kJ/mol with respect to MIN1 (and 15 kJ/mol above the product asymptote). Alternatively, the cyclic cofragment ^1^*c*-CH_2_(N)CCN is formed (channel (d)). In this case, the TS3 transition state, which is located at a somewhat higher energy, that is, +215 kJ/mol with respect to MIN1 and +24 kJ/mol above the product asymptote, must be overcome. The reaction enthalpy for the two channels is −236 and −223 kJ/mol, respectively.

MIN1 can also isomerize to other intermediates. The migration of an H atom from C_1_ to the N atom of the C(N)C ring leads, via TS1, to the formation of the intermediate MIN3, located at 367 kJ/mol below the reactant energy asymptote. MIN3 can dissociate by the fission of the N–H bond forming the ^1^*c*-CH(N)CHCN coproduct and is, therefore, an additional route to channel (e). MIN3 can also isomerize (through TS20) to another intermediate (MIN2) located 522 kJ/mol below the reactant energy asymptote. Two subsequent isomerization processes, characterized by the presence of small barriers, can lead to the formation of MIN21 and MIN9, which show different orientations of the newly formed NH group. Once formed, both MIN9 and MIN21 can undergo dissociation through the two transition states TS37 and TS43, respectively, leading to the formation of H + *cis*-^1^HNCCHCN (cyanoketenimine, channel (g)). MIN2 can also dissociate into *trans*-^1^HNCCHCN (channel (h)). This is the most exothermic channel (enthalpy change −319 kJ/mol) among those starting with the addition of N(^2^D) to the π bond of vinyl cyanide, but a high barrier of 226 kJ/mol (from MIN2) must be overcome to reach TS45 (the exit barrier height with respect to products is +23 kJ/mol).

MIN1 can directly undergo a ring-opening process associated with breaking of the C_1_–C_2_ bond, leading to the formation of the linear intermediate MIN12, located 499 kJ/mol below the reactant energy asymptote. This isomerization step features a barrier of 130 kJ/mol (from MIN1) associated with the TS21 transition state (see Figures 1 and 7). Also in this case, two possible linear molecular products can be formed, depending on which C–H bond undergoes fission. The elimination of an H atom from the terminal (C_1_) carbon atom leads to the formation of the ^1^CHNCHCN coproduct (reaction enthalpy of −200 kJ/mol, channel (c)). Alternatively, the H-elimination from the C_2_ atom leads to the formation of ^1^CH_2_NCCN (channel (b), reaction enthalpy of −195 kJ/mol). Both channels are characterized by exit barriers (corresponding to TS47 and TS48, respectively) located at an energy very similar to that of the related product asymptote.

One last isomerization from the MIN1 intermediate is possible, leading to the formation of another cyclic intermediate, MIN5, located at 333 kJ/mol below the reactant energy asymptote. The TS6 transition state from MIN1 to MIN5 is associated with the transfer of an H atom from the C_2_ carbon to the C_3_ carbon atom (of the CN group).

Finally, the C2–C3 bond fission in MIN1 can lead to the formation of CN + ^1^*c*-CH_2_(N)CH (channel (a)) in a barrierless process. The C2–C3 bond fission in MIN5 is responsible for the formation of HCN + *c*-CH_2_(N)C with an exothermicity of 267 kJ/mol. The barrier of 111 kJ/mol (from MIN5) must be overcome, and the related transition state TS9 clearly shows the weakening of a C2–C3 bond with the C–C distance going from 1.461 Å in MIN5 up to 2.147 Å (see the Supporting Information).

#### 4.1.2 Reactive Pathways Starting with the N(^2^D) Addition to the Triple Bond or Nitrogen Lone Pair of the Nitrile Group

The reaction pathways analyzed in the previous section are common to all the organic molecules with a double C–C bond. However, in the case of vinyl cyanide, N(^2^D) can attack the triple bond or the N lone pair of the nitrile group. This is a peculiar observation because, in most cases, the cyano group is considered as a pseudohalogen; that is, its triple bond confers a large stability to the cyano group, and it normally behaves as a spectator. Nevertheless, the energy content of the electronically excited state of nitrogen atoms is so high that a chemical attack on the CN group is also feasible. In Figure 2, we have reported the reactive pathways originating from both the addition to the triple bond of CN or to the lone pair located on the N atom of the CN group.

The addition to the lone pair of the N atom is characterized by a very small barrier (TSi_2_, located at +0.3 kJ/mol at the present level of calculations) and leads to the formation of MIN22, which lies 343 kJ/mol under the reactants. A bridge attack to the triple bond leads to the cyclic intermediate MIN17, which lies 333 kJ/mol below the reactants. A submerged transition state located 5 kJ/mol below the reactants is present, implying the presence of an initial van der Waals adduct that we were not able to localize at the B3LYP7aug-cc-pVTZ level of calculation. MIN17 can isomerize to MIN22 through a barrier of 180 kJ/mol or to MIN23 with a barrier height of 321 kJ/mol or directly dissociate to the products ^1^c-CH_2_CCNN + H in a weakly exothermic (Δ*H*_0_^0^ = −30 kJ/mol) channel or to ^1^c-CNN + CH_2_CH in an even less exothermic (Δ*H*_0_^0^ = −2 kJ/mol) channel.

Once formed, MIN22 can isomerize to MIN15, which is less stable by 49 kJ/mol, through a barrier of 195 kJ/mol or to MIN18, which is slightly more stable than MIN22 (by 2 kJ/mol), by overcoming an energy barrier of 240 kJ/mol. MIN22 can also dissociate through a barrier of 47 kJ/mol to the products CH_2_CCH + N_2_ (channel (j)), which represents the most exothermic of all reaction channels (Δ*H*_0_^0^ = −522 kJ/mol) of the N(^2^D) + C_2_H_3_CN reaction. MIN23 can isomerize to the more stable (by 139 kJ/mol) MIN18 through a barrier of only 1 kJ/mol. MIN18 can dissociate to ^1^CH_2_CCNN + H in a channel that is exothermic by 105 kJ/mol or to ^1^CCNNC + CH_3_ in a channel that is exothermic by 72 kJ/mol. Both these exit channels are barrierless. Finally, MIN18 can also dissociate through a barrier of 53 kJ/mol to CH_3_CC + N_2_, which is a strongly exothermic channel (Δ*H*_0_^0^ = −344 kJ/mol) (channel (i)).

MIN15 can dissociate to ^1^c-C_3_H_2_N_2_ + H in an almost thermoneutral process by overcoming an energy barrier of 312 kJ/mol. This saddle point, TS44, lies above the reactants (+18 kJ/mol). MIN15 can also isomerize to the cyclic isomer MIN10 (more stable by 111 kJ/mol) through a barrier of 96 kJ/mol. MIN10 can dissociate to ^1^c-C_3_H_2_N_2_ + H (Δ*H*_0_^0^ = 0 kJ/mol) in a barrierless process or isomerize to MIN9, which is more stable by 115 kJ/mol, through a barrier of 77 kJ/mol. MIN9 is a species in common with the scheme shown in Figure 1 and is then a way to join the two schemes.

### 4.2 RRKM Calculations of the Product Branching Fractions

RRKM calculations of product BFs were performed considering the collision energy of the CMB experiment (31.4 kJ/mol) and at three different temperatures corresponding to the surface temperature of Titan (94 K), its stratospheric temperature (175 K), and room temperature (298 K). The calculations were performed starting from MIN1, that is, the addition intermediate formed by the attack of N(^2^D) to the double bond of C_2_H_3_CN. We did not consider the alternative initial attacks because the formation of MIN1 (see Figure 1) is much more competitive than the formation of MIN17 and MIN22 (see Figure 2), the formation of which is preceded by the presence of transition states at an energy close to that of the reactants. This certainly reduces the reactive flux. In addition to that, the favored minimum energy path from MIN17 and MIN22 is the one leading to N_2_ formation accompanied by the formation of the propargyl radical or its isomer (CH_2_CCH or CH_3_CC). As already mentioned, we could not detect a reactive signal at either *m*/*z* = 39 or 38. This implies that the contribution from the attack to the nitrile group is expected to be minor.

The resulting BFs, considering only the expectedly dominant N(^2^D) addition to the double bond of vinyl cyanide, are shown in Table 2. Once MIN1 is formed (see Figure 1), it can undergo either isomerization to the linear structure MIN12 or it can directly dissociate to form both H + ^1^*c*-CH_2_(N)CCN and H + ^1^*c*-CH(N)CHCN (channels (d) and (e), respectively). According to the present RRKM calculations, the main reaction channel is associated with the formation of H + ^1^*c*-CH(N)CHCN (channel (e)). This is the dominant channel at *E*_c_ = 31.4 kJ/mol (BF = 35%) and the second most important channel under the conditions of relevance in Titan (BF = 29.9%). The dominant channel under the conditions of Titan (175 K) is channel (c) (BF = 32.2%) associated with the fission of the C–H bond of the linear intermediate MIN12, leading to H + ^1^CHNCHCN. Also, the other channel originating from MIN12 (H + ^1^CH_2_NCCN, channel (b)) has a comparable value of BF (28.9%). H-elimination from the C_2_ carbon of the cyclic intermediate MIN1 represents the fourth most important channel (d) (with BF = 7.4% at *E*_c_ = 31.4 kJ/mol and 5.8% at 175 K).

**Table 2:** Statistical Branching Fractions (%) for the Main Product Channels of the Reaction N(^2^D) + CH_2_CHCN (from N(^2^D) Addition to the Double Bond) at Three Temperatures (*T’s*) and at the *E*_c_ of the CMB Experiment

reaction channel	products	*T* = 94 K	*T* = 175 K	*T* = 298 K	*E*_c_ = 31.4 kJ/mol
a	^1^*c*-CH_2_(N)CH + CN	0.02%	0.02%	0.02%	0.02%
b	^1^CH_2_NCCN + H	29.3%	28.9%	28.3%	26.3%
c	^1^CHNCHCN + H	32.8%	32.2%	31.1%	28.1%
d	^1^*c*-CH_2_(N)CCN + H	5.6%	5.8%	6.2%	7.4%
e	^1^*c*-CH(N)CHCN + H	29.3%	29.9%	31.4%	35.0%
f	^1^*c*-CH_2_(N)C + HCN	0.1%	0.1%	0.1%	0.2%
g	*cis*-^1^HNCCHCN + H	1.2%	1.2%	1.3%	1.3%
h	*trans*-^1^HNCCHCN + H	1.6%	1.6%	1.6%	1.6%

Finally, the products H + *cis*-^1^NHCCHCN and H + *trans*-^1^HNCCHCN (channels (g) and (h), respectively), originating from MIN2 and MIN9, are statistically predicted to account, respectively, for only 1.3% and 1.6% of the total product yield at the *E*_c_ of the experiment. The C–C bond fission of the intermediates MIN3 and MIN5, leading to CN + ^1^*c*-CH_2_(N)CH and HCN + ^1^*c*-CH_2_(N)C (channels (a) and (f)), appear to be much less probable with values of BFs lower than 1% (0.02% and 0.2%, respectively).

Overall, the main reaction channels under all conditions considered are (1b), (1c), and (1e) each accounting for ca. 30% of the global reactive flux (see Table 2). The other H-displacement channels and C–C bond breaking channels account only for a small part of the reactive flux. The theoretical results indicate that the product BFs exhibit some (weak) temperature dependence (see Table 2).

We wish to emphasize that the experimental results indicate that the dominant product channels of the N(^2^D) + CH_2_CHCN reaction at *E*_c_ = 31.4 kJ/mol are isomeric H-displacement channels and that the CN, HCN, and N_2_ forming channels are negligible. The relative importance of the isomeric H channels cannot be disentangled experimentally because of their similar exothermicity and formation micromechanism. Interestingly and most notably, the statistical calculations on the theoretical reaction PES fully corroborate the experimental finding that only H-displacement channels are formed and afford a quantitative prediction of their relative importance (see Table 2).

## 5 Discussion

The H-displacement channels leading to the isomeric products ^1^CH_2_NCCN (channel (b)), ^1^CHNCHCN (channel (c)), and ^1^*c*-CH(N)CHCN (channel (e)) are theoretically predicted to be dominant, either under the conditions of our CMB experiments or under the conditions of the surface and stratosphere of Titan (see Table 2). As already mentioned, given the similarity of the reaction exothermicities, the same combination of masses, and similarity in the reaction mechanism, during the analysis of the CMB data, we have not been able to disentangle the contributions of the possible isomeric C_3_H_2_N_2_ product species to the reactive signal. However, the experimental results, and in particular the shape of the CM angular distribution, indicate that the title reaction proceeds with the formation of a covalently bound reaction intermediate, and this well justifies the use of a statistical approach to derive the product branching fractions.

The C_3_H_2_N_2_ product CM angular distribution (Figure 5 (top)) exhibits constant intensity throughout the angular range: the lack of polarization (or limited polarization considering the error bars) indicates a high rotational excitation of the products, on the basis of the conservation of the total angular momentum.^94−96^ The symmetry of the angular distribution is typical of a mechanism that proceeds through the formation of a long-lived complex that does not retain the memory of the initial direction of the reagents; hence, the products are scattered symmetrically throughout the space. This is fully supported by our calculations of the PES, which is characterized by bound intermediates associated with deep wells along the possible reaction pathways (see Figure 1).

The reaction mechanism derived for the title reaction is in line with the one already characterized for N(^2^D) + C_2_H_4_. We can directly compare the CMB results for both systems since our research group has studied the latter reaction using the same technique under similar experimental conditions and the same theoretical approach.^29^ Also, in that case, the H-displacement channels (products detected at *m*/*z* = 41 and 40, corresponding to parent and daughter ions of the product with gross formula C_2_H_3_N, respectively) were found to be dominant at two different collision energies (17.2 and 28.4 kJ/mol). As already mentioned, in the present experiment, it was not possible to measure a full set of angular and velocity distributions at the mass of the parent radical product with gross formula C_3_H_2_N_2_ (*m*/*z* = 66), because the S/N was too low (due to high dissociative ionization of the parent ion). The LAB angular distributions for the two different systems are both bell-shaped around the CM angle, and both are isotropic and/or slightly polarized (within the error bands) in the CM frame, which indicates that both reactions proceed via formation of a long-lived complex. The product translational energy distributions also show interesting similarities with a similar fraction of the total available energy converted into product translational energy. These similarities are fully justified when we consider the PES of the two reactions. Notably, the number of channels for the title reaction has significantly increased compared to the reaction with ethylene because the presence of a CN group removes the symmetry of ethylene. Nevertheless, the energies associated with the PES minima of C_2_H_4_N are very similar to those associated with the PES of C_3_H_3_N_2_.

With the comparison of the two PESs, it can be seen that the channel leading to ^1^c-CH_2_(N)CH + H for N(^2^D) + ethylene is equivalent for the two reaction channels ^1^*c*-CH_2_(N)CCN + H (d) and 1*c*-CH(N)CHCN + H (e) on the PES of the title reaction, while the channel leading to ^1^CH_2_NCH + H in N(^2^D) + C_2_H_4_ is equivalent to the reaction channels (b) and (c), leading to ^1^CH_2_NCCN + H (b) and ^1^CHNCHCN + H (c). The similarities of the topology of the two PESs are mirrored in the values of the RRKM BFs for the two systems. In the case of the N(^2^D) + ethylene reaction, the two most important channels are those leading to CH_2_(N)CH (2*H*-azirine) + H and CH_2_NCH + H with BFs (at 28.2 kJ/mol) of 28.6% and 59.6%, respectively. In the case of the N(^2^D) + vinyl cyanide reaction, the sum of the BFs of channels (b) and (c) is equal to 54.4% in line with the CH_2_NCH + H channel of N(^2^D) + C_2_H_4_. On the other hand, the sum of the BFs of the reaction channels (d) and (e) is 42.5%, slightly larger than the one associated with the cyclic product CH_2_(N)CH + H. Another similarity between the two systems can be seen when evaluating the variations of the BFs of the reaction products as a function of temperature/collision energy. For both reactions, as the temperature/collision energy decreases, the BFs of the linear products that are formed in H-displacement channels tend to increase, while the BF of cyclic products slightly decreases.

## 6 Implications for the Atmosphere of Titan

As already discussed in Section 1, the title reaction has been included in the recent photochemical models of Titan’s atmosphere. Loison et al.^23^ were the first to consider it and, by analogy with the reaction N(^2^D) + C_2_H_4_, they introduced an H-displacement channel, leading to HNCCHCN + H with an estimated rate coefficient of 2.3 × 10^–10^ exp(−503/*T*) cm^3^ molec^–1^ s^–1^. In addition, they included a reaction channel leading to N_2_ + C_3_H_3_ with an estimated rate coefficient of 4 × 10^–11^ cm^3^ molec^–1^ s^–1^ by analogy with the reaction N(^2^D) + HCN. In a more recent model, Vuitton et al.,^19^ instead, included the title reaction as N(^2^D) + C_3_H_3_N → H + C_3_H_2_N_2_, making no distinction between the possible isomeric products having the same empirical formula C_3_H_2_N_2_. Also in this case, an estimated rate coefficient of 2.3 × 10^–10^ exp(−503/*T*) cm^3^ molec^–1^ s^–1^ was employed. In both models, it is not clear which destiny HNCCHCN or C_3_H_2_N_2_ has.

First, according to our experimental and theoretical results, the suggestion that N_2_ + C_3_H_3_ is the main reaction channel is not confirmed. We have not observed its occurrence in the CMB experiments, and the electronic structure calculations of the PES clearly show that these channels are not competitive, especially under the conditions of Titan. However, it is worth noting that this reaction has the capability of destroying the −CN moiety, even though the cyano group is often referred to as a *pseudohalogen* for its strong chemical bond. At high temperatures, the N_2_ formation channel can become competitive.

Second, according to our RRKM estimates, the title reaction leads to the formation of three main isomeric C_3_H_2_N_2_ product channels with comparable BFs of about 30%, ^1^CHNCHCN + H (BF = 32.2%), ^1^*c*-CH(N)CHCN + H (BF = 29.9%), and ^1^CH_2_NCCN + H (BF = 28.9%), under the conditions of Titan’s stratosphere (175 K). All the other channels originating from the MIN1 intermediate (see Figure 1) are essentially negligible. Even though it has not been explicitly stated, the HNCCHCN isomer used by Loison et al.^23^ is cyanoketenimine, that is, the molecular product that is formed in channels (g) and (h) in its *cis*- and *trans*-forms. The yield of these two channels is, however, very small being ca. 1%. Another aspect that needs to be considered is that the rate coefficient employed in the models by Loison et al.^23^ and Vuitton et al.^19^ is based on the N(^2^D) + C_2_H_4_ rate coefficient as it was estimated by Sato et al.^97^ in a small temperature range (from 230 to 292 K). However, recent CRESU experiments have demonstrated that the rate coefficient is almost independent of *T* in the range between 50 and 296 K with values around 7 ÷ 9 × 10^–11^ cm^3^ s^–1^.^98^ Notably, at the temperature of the stratosphere of Titan, the resulting rate coefficient is almost seven times larger^98^ than that used in the models. The experimental data are corroborated by the theoretical investigation of the entrance channel at a high level of calculations, which confirms that there is not an entrance barrier.

Therefore, we also expect that for the title reaction the rate coefficient will be close to the gas kinetics limit, around 10^–10^ cm^3^ s^–1^, and that the reaction will compete with the other destruction mechanisms of vinyl cyanide mentioned in Section 1 because of the significant abundance of N(^2^D) above 1000 km. In addition, we suggest that the main molecular products ^1^CHNCHCN, ^1^*c*-CH(N)CHCN, and ^1^CH_2_NCCN be considered in the photochemical models. They are closed-shell species but either are highly unsaturated or have a strained 3-membered ring. Therefore, they can easily react and contribute to the growth of the N-rich organic molecules, which appear to be the main constituents of the orange haze that covers this exotic moon of Saturn.^6,9,13,99^ The potential importance of these N-containing chemical species in prebiotic chemistry has been extensively discussed.^5,8,9,99−101^

## 7 Conclusions

The N(^2^D) reaction with vinyl cyanide has been investigated by the CMB technique with mass spectrometric detection at the collision energy of 31.4 kJ/mol, and electronic structure calculations of the underlying potential energy surface have been carried out. The angular and TOF distributions of the C_3_H_2_N_2_ products in the LAB frame along with the derived CM best-fit functions indicate that one or more H-displacement channels are open and that one or more C_3_H_3_N_2_ long-lived intermediates are formed. No evidence of CN, HCN, and N_2_ forming channels was observed experimentally. RRKM statistical calculations on the doublet C_3_H_3_N_2_ PES allow one to discriminate the possible isomeric H product channels formed in the experiments as well as those formed under the conditions relevant for the atmosphere of Titan. Ten main competing channels (six H-displacement channels, one CN and one HCN forming channel, two N_2_ forming channels) have been identified theoretically. Among them, three of the possible H-displacement channels (those leading to ^1^CH_2_NCCN, ^1^CHNCHCN, and ^1^*c*-CH(N)CHCN) each feature a BF of about 30%. Overall, at *E*_c_ = 31.4 kJ/mol, the theory predicts that the H-displacement channels account for 99.7% of the reactive flux, and this large fraction remains essentially the same down to about 94 K (Table 2). The main H-forming channels all arise from the N(^2^D) addition to the double bond of CH_2_CHCN (see Figure 1).

The reaction is theoretically found to be barrierless, which suggests a nearly gas kinetic rate constant down to the low temperatures of Titan’s atmosphere, in line with recent kinetic results (by the CRESU technique) on the related N(^2^D) + C_2_H_4_ reaction,^98^ which was found to be about seven times faster than that assumed in models.

Our studies provide the first evidence that the reaction of N(^2^D) with CH_2_CHCN is a potential pathway to produce, in the conditions of the atmosphere of Titan, molecular species, which in turn can further react efficiently with other species acting as precursors of other nitriles or more complex organic molecules containing two CN bonds by consecutive reactions.

In conclusion, our findings on the title reaction could be incorporated into photochemical models of N_2_-rich planetary atmospheres (in particular of Titan) bearing a significant amount of unsaturated molecules, such as vinyl cyanide, and might also contribute to the re-evaluation of the role of gas-phase neutral chemistry in heavily UV irradiated interstellar environments containing N_2_ and vinyl cyanide.

## Supporting Information Available

List of all possible exothermic combinations of products identified for the N(^2^D) + CH_2_CHCN reaction, determined at the CCSD(T)/aug-cc-pVTZ//B3LYP/aug-cc-pVTZ level of theory; reaction enthalpies and barrier heights (kJ/mol, 0 K) computed at the CSD(T)/aug-cc-pVTZ level of theory, considering the geometries obtained at the B3LYP/aug-cc-pVTZ level, for dissociation and isomerization processes for the system N(^2^D) + CH_2_CHCN (PDF)
