# Supplementary material for: The N(2D) + CH2CHCN (Vinyl Cyanide) Reaction: A Combined Crossed Molecular Beam and Theoretical Study and Implications for the Atmosphere of Titan
Source: J Phys Chem A. 2022 Sep 2;126(36):6110–23. doi: 10.1021/acs.jpca.2c04263 (PMC9483977; doi:10.1021/acs.jpca.2c04263)
Supplement: Supplementary file 1 — jp2c04263_si_001.pdf [file jp2c04263_si_001.pdf]

Supporting Information (SI) for the paper

The N(^{2}D) + CH_2CHCN (Vinyl Cyanide) Reaction: A Combined Crossed Molecular Beam and Theoretical Study and Implications for the Atmosphere of Titan

Gianmarco Vanuzzo,[a] Demian Marchione,[a] Luca Mancini,[a] Pengxiao Liang,[a] Giacomo Pannacci,[a] Pedro Recio,[a], Yuxin Tan,[a] Marzio Rosi,[b] Dimitrios Skouteris,[c] Piergiorgio Casavecchia,[a*] and Nadia Balucani[a*]

[a] Dipartimento di Chimica, Biologia e Biotecnologie, Università degli Studi di Perugia, 06123 Perugia, Italy

[b] Dipartimento di Ingegneria Civile e Ambientale, Università degli Studi di Perugia, 06100 Perugia, Italy

[c] Master-Tec srl, Via Sicilia 41, 06128 Perugia, Italy

Table S1: List of all possible exothermic combinations of products identified for the N(^{2}D) + CH_2CHCN reaction, determined at the CCSD(T)/aug-cc-pVTZ//B3LYP/aug-cc-pVTZ level of theory.

N(^{2}D) + CH_2CHCN	→	^{1}*c*-$C_3H_2N_2$ + H	- 0.04 kJ/mol
	→	^{1}*c*-C(N)N + CH_2CH	- 2 kJ/mol
	→	^{3}NCCN + CH_3	- 5 kJ/mol
	→	H_2CN + ^{1}CCNH	- 7 kJ/mol
	→	H_2CN + ^{3}CCNH	- 11 kJ/mol
	→	^{3}NCHCCNH + H	- 12 kJ/mol
	→	^{3}*c*-CH(NH)CH + CN	- 17 kJ/mol
	→	^{1}*c*-CH(N)CCHN + H	- 23 kJ/mol
	→	^{1}*c*-CH_2CC(N)N + H	- 30 kJ/mol
	→	^{1}*c*-CH_2NC(N)C + H	- 47 kJ/mol
	→	^{1}*c*-CH_2(NCN)C + H	- 48 kJ/mol
	→	HCNH + ^{1}HCCN	- 54 kJ/mol
	→	^{1}CHNCCNH + H	- 61 kJ/mol
	→	^{1}*c*-CH(N)CCNH + H	- 68 kJ/mol
	→	^{1}*c*-CH(NCN)CH + H	- 70 kJ/mol
	→	$^3CH_2$NCCN + H	- 70 kJ/mol
	→	^{1}CCNN + CH_3	- 72 kJ/mol
	→	^{3}CHNCHCN + H	- 87 kJ/mol
	→	^{1}NHCHCCN-i + H	- 90 kJ/mol
	→	^{3}CHNHCCN + H	- 91 kJ/mol
	→	*c*-CH(NH)C + ^{1}HNC	- 93 kJ/mol
	→	^{1}*c*-CH(NH)CCN + H	- 98 kJ/mol
	→	HCNH + ^{3}HCCN	- 104 kJ/mol
	→	$^1CH_2$CCNN + H	- 105 kJ/mol
	→	$^1CH_2$NH + C_2N	- 106 kJ/mol
	→	^{1}HCCN + H_2CN	- 108 kJ/mol
	→	^{1}*c*-C(NH)CHCN + H	- 116 kJ/mol

→	H_2CN + CCNH	- 119 kJ/mol
→	*c*-CH(NH)C + ^{1}HCN	- 150 kJ/mol
→	^{1}c-CH_2(N)CH + CN	- 153 kJ/mol
→	^{3}NCHCHCN + H	- 154 kJ/mol
→	^{3}HCCN + H_2CN	- 157 kJ/mol
→	^{3}CHNCHCN + H	- 161 kJ/mol
→	$^1CH_2NCCN$ + H	- 194 kJ/mol
→	^{1}CHNCHCN + H	-200 kJ/mol
→	c-CH_2(N)C + ^{1}HNC	- 210 kJ/mol
→	^{1}NHCCCNH + H	- 218 kJ/mol
→	^{1}*c*-CH_2(N)CCN + H	- 223 kJ/mol
→	^{1}*c*-CH(N)CHCN + H	- 236 kJ/mol
→	c-CH_2(N)C + ^{1}HCN	- 267 kJ/mol
→	^{1}NHCCHCN + H	- 318(.8) kJ/mol
→	^{1}HNCCHCN + H	- 319(.2) kJ/mol
→	CH_3CC + N_2	- 344 kJ/mol
→	^{3}CNCHCN + H_2	- 365 kJ/mol
→	CH_2NC + ^{1}HCN	- 382 kJ/mol
→	^{1}NCCN + CH_3	- 420 kJ/mol
→	CH_2CCH + N_2	- 516 kJ/mol

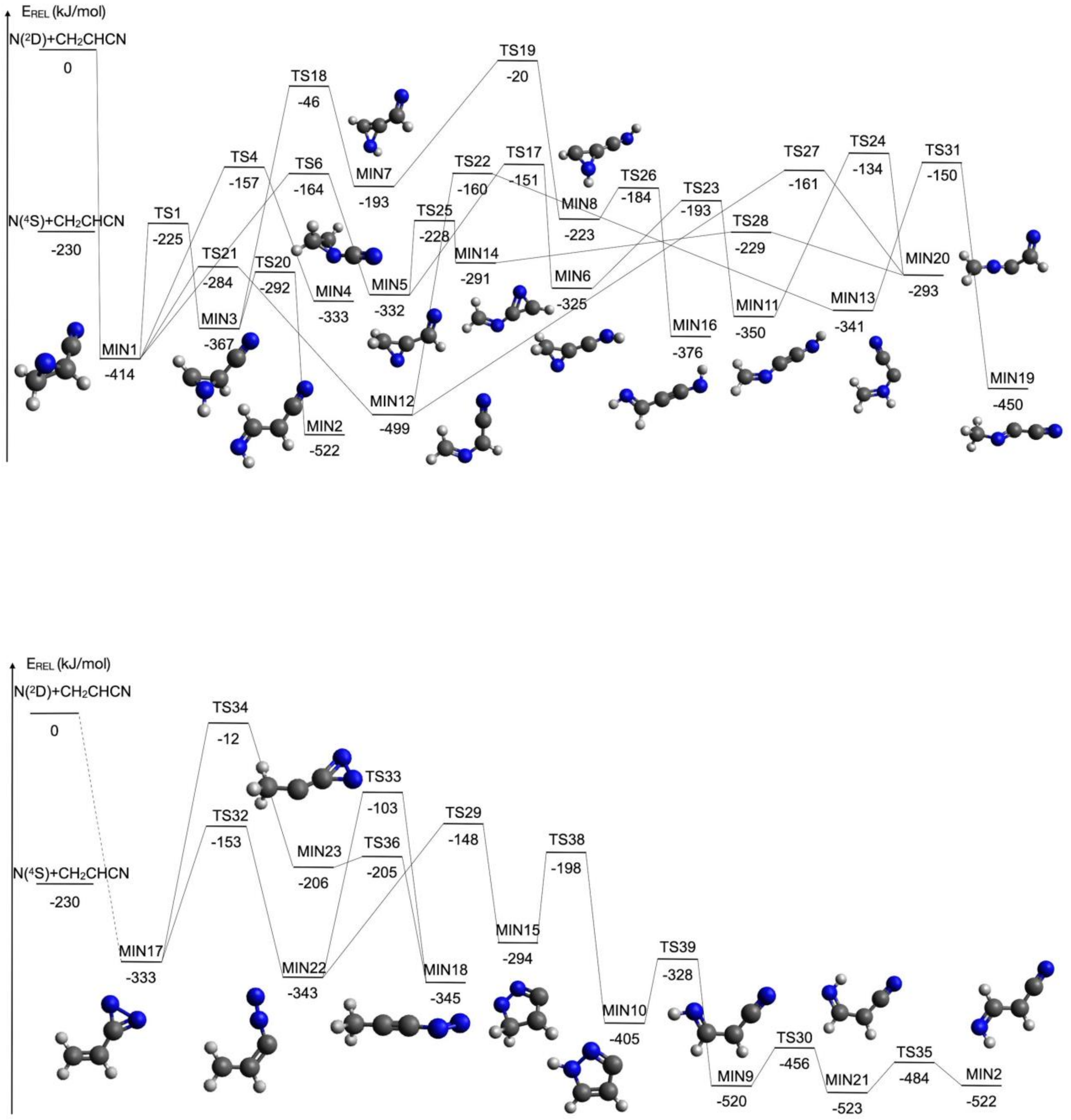

Figure S1: Schematic representation of the Potential Energy Surface including all possible isomerization processes for the $N(^2D)$ + CH_2CHCN reaction identified at the CCSD(T)/aug-cc-pVTZ//B3LYP/aug-cc-pVTZ level of theory. (Top): considering only $N(^2D)$ addition to the double bond. (Bottom): considering the attack of the $N(^2D)$ atom to the triple bond of CN and to the lone pair of the nitrogen atom.

List of all possible products starting from the various minima identified in the Potential Energy Surface, with relative energies reported at the CCSD(T)/aug-cc-pVTZ//B3LYP/aug-cc-pVTZ level of theory.

MIN1

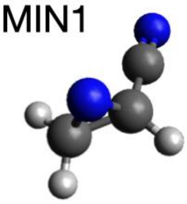

E_{REL}= - 414 kJ/mol

- ⟶ ^{1}c-CH_2(N)CH + CN — E_{REL}= - 153 kJ/mol
- ⟶ TS3 ⟶ ^{1}c-CH_2(N)CCN + H — E_{REL}= - 223 kJ/mol
- ⟶ TS2 ⟶ ^{1}c-CH(N)CHCN + H — E_{REL}= - 236 kJ/mol

MIN3

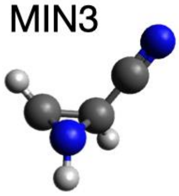

E_{REL}= - 367 kJ/mol

- ⟶ ^{1}c-CH(NH)CH + CN — E_{REL}= -17 kJ/mol
- ⟶ TS7 ⟶ ^{1}c-CH(NH)CCN + H — E_{REL}= - 98 kJ/mol
- ⟶ ^{1}c-C(NH)CHCN + H — E_{REL}= - 116 kJ/mol
- ⟶ TS5 ⟶ ^{1}c-CH(N)CHCN + H — E_{REL}= - 236 4kJ/mol

MIN4

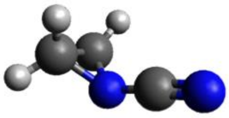

E_{REL}= - 333 kJ/mol

- ⟶ ^{1}c-CH_2(NCN)C + H — E_{REL}= - 40 kJ/mol
- ⟶ TS8 ⟶ ^{1}c-CH(NCN)CH + H — E_{REL}= - 70 kJ/mol
- ⟶ ^{1}c-CH_2(N)CH + CN — E_{REL}= - 153 kJ/mol

MIN5

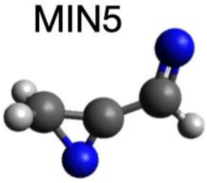

E_{REL}= - 333 kJ/mol

- ⟶ ^{1}c-CH(N)CCHN + H — E_{REL}= - 23 kJ/mol
- ⟶ TS11 ⟶ ^{1}c-CH_2(N)CCN + H — E_{REL}= - 223 kJ/mol
- ⟶ TS9 ⟶ c-CH_2(N)C + ^{1}HCN — E_{REL}= - 267 kJ/mol

MIN6

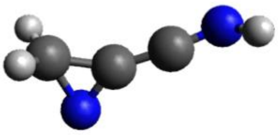

E_{REL}= - 325 kJ/mol

- ⟶ TS13 ⟶ ^{1}c-CH(N)CCNH + H — E_{REL}= - 68 kJ/mol
- ⟶ TS12 ⟶ c-CH_2(N)C + ^{1}HNC — E_{REL}= - 210 kJ/mol
- ⟶ TS14 ⟶ ^{1}c-CH_2(N)CCN + H — E_{REL}= - 223 kJ/mol

MIN7

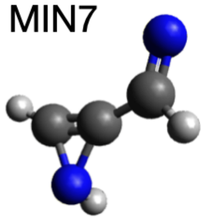

E_{REL}= - 193 kJ/mol

⟶	^{1}c-CH(N)CCHN + H	E_{REL}= - 23 kJ/mol
⟶	^{1}c-CH(NH)CCN + H	E_{REL}= - 98 kJ/mol
⟶ TS10 ⟶	c-CH(NH)C + ^{1}HCN	E_{REL}= - 150 kJ/mol

MIN8

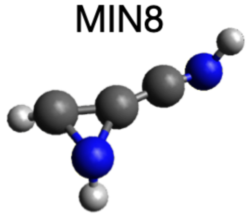

E_{REL}= - 223 kJ/mol

⟶ TS15 ⟶	^{1}c-CH(N)CCNH + H	E_{REL}= - 68 kJ/mol
⟶	c-CH(NH)C + ^{1}HNC	E_{REL}= - 93 kJ/mol
⟶ TS16 ⟶	^{1}c-CH(NH)CCN + H	E_{REL}= - 98 kJ/mol

MIN14

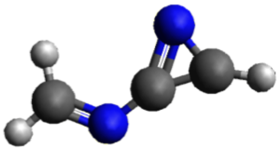

E_{REL}= - 291 kJ/mol

⟶	^{1}c-CH_2NC(N)C + H	E_{REL}= - 47 kJ/mol
⟶	CH_2N + ^{1}c-C(N)CH	E_{REL}= - 119 kJ/mol

MIN2

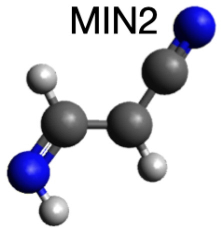

E_{REL}= - 522 kJ/mol

⟶	^{1}HCCN + HCNH	E_{REL}= - 54 kJ/mol
⟶	^{3}CHNHCCN + H	E_{REL}= - 91 kJ/mol
⟶	^{3}HCCN + HCNH	E_{REL}= - 104 kJ/mol
⟶	^{3}CHNCHCN + H	E_{REL}= - 161 kJ/mol
⟶ TS45 ⟶	^{1}HNCCHCN + H	E_{REL}= - 319 kJ/mol

MIN21

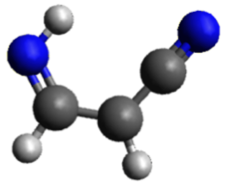

E_{REL}= - 523 kJ/mol

⟶	^{1}HCCN + HCNH	E_{REL}= - 54 kJ/mol
⟶	^{3}CHNHCCN + H	E_{REL}= - 91 kJ/mol
⟶	^{3}HCCN + HCNH	E_{REL}= - 104 kJ/mol
⟶	^{3}NCHCHCN + H	E_{REL}= - 154 kJ/mol
⟶ TS43 ⟶	^{1}NHCCHCN+ H	E_{REL}= - 319 kJ/mol

MIN12

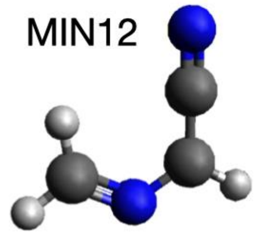

E_{REL}= - 499 kJ/mol

→	$^{3}CH_2NCCN + H$	E_{REL}= - 70 kJ/mol
→	$^{3}CHNCHCN + H$	E_{REL}= - 87 kJ/mol
→	$CH_2N + {}^{1}HCCN$	E_{REL}= - 108 kJ/mol
→	$CH_2N + {}^{3}HCCN$	E_{REL}= - 157 kJ/mol
→ TS47 →	$^{1}CH_2NCCN + H$	E_{REL}= - 194 kJ/mol
→ TS48 →	$^{1}CHNCHCN + H$	E_{REL}= - 200 kJ/mol
→ TS49 →	$^{1}CNCHCN + H_2$	E_{REL}= - 365 kJ/mol

MIN11

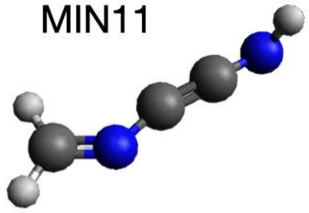

E_{REL}= - 350 kJ/mol

→	$CH_2N + {}^{1}CCNH$	E_{REL}= - 7 kJ/mol
→	$CH_2N + {}^{3}CCNH$	E_{REL}= - 11 kJ/mol
→	$^{1}CHNCCNH + H$	E_{REL}= - 61 kJ/mol
→	$^{3}CH_2NCCN + H$	E_{REL}= - 70 kJ/mol
→	$^{1}CH_2NCCN + H$	E_{REL}= - 194 kJ/mol

MIN9

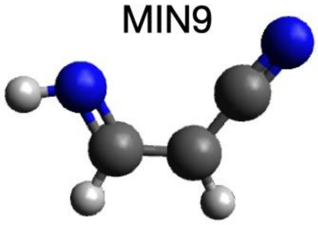

E_{REL}= - 520 kJ/mol

→	$^{1}HCCN + HCNH$	E_{REL}= - 54 kJ/mol
→	$^{3}NHCHCCN$-i+ H	E_{REL}= - 90 kJ/mol
→	$^{3}HCCN + HCNH$	E_{REL}= - 104 kJ/mol
→	$^{3}NCHCHCN + H$	E_{REL}= - 154 kJ/mol
→ TS37 →	$^{1}NHCCHCN + H$	E_{REL}= - 319 kJ/mol

MIN16

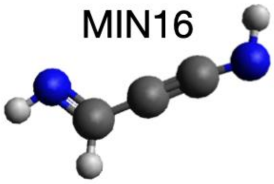

E_{REL}= - 376 kJ/mol

→	$^{3}NCHCCNH + H$	E_{REL}= - 12 kJ/mol
→	$^{3}NHCHCCN$-i + H	E_{REL}= - 90 kJ/mol
→	$^{1}NHCCCNH + H$	E_{REL}= - 217 kJ/mol

MIN20

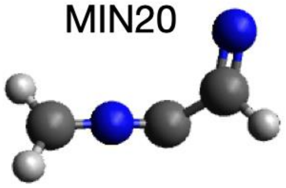

E_{REL}= - 293 kJ/mol

→	$^{3}CH_2NCCN + H$	E_{REL}= - 70 kJ/mol
→	$^{1}CH_2NCCN + H$	E_{REL}= - 194 kJ/mol
→ TS46 →	$CH_2NC + {}^{1}HCN$	E_{REL}= - 382 kJ/mol

MIN13

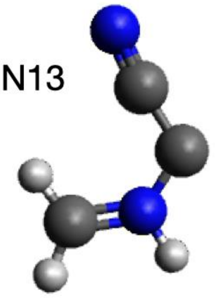

E_{REL}= - 341 kJ/mol

→ $^{3}CH_2NCCN$ + H E_{REL}= - 70 kJ/mol
→ $^{1}CH_2NH$ + CCN E_{REL}= - 106 kJ/mol
→ $^{1}CH_2NCCN$ + H E_{REL}= - 194 kJ/mol

MIN19

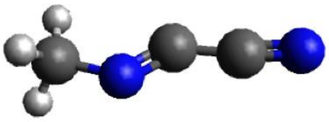

E_{REL}= - 450 kJ/mol

→ $^{3}NCCN$ + CH_3 E_{REL}= - 5 kJ/mol
→ $^{3}CH_2NCCN$ + H E_{REL}= - 70 kJ/mol
→ $^{1}CH_2NCCN$ + H E_{REL}= - 194 kJ/mol
→ $^{1}NCCN$ + CH_3 E_{REL}= - 420 kJ/mol

MIN18

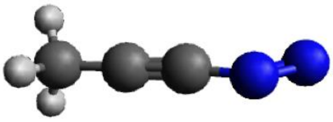

E_{REL}= - 354 kJ/mol

→ $^{1}CCNN$ + CH_3 E_{REL}= - 72 kJ/mol
→ $^{1}CH_2CCNN$ + H E_{REL}= - 105 kJ/mol
→ TS40 → CH_3CC + $^{1}N_2$ E_{REL}= - 344 kJ/mol

MIN10

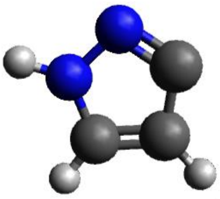

E_{REL}= - 405 kJ/mol

→ $^{1}c\text{-}C_3H_2N_2$+ H E_{REL}= - 0.04 kJ/mol

MIN15

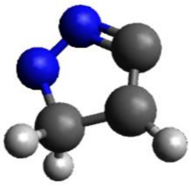

E_{REL}= - 294 kJ/mol

→ TS44 → $^{1}c\text{-}C_3H_2N_2$+ H E_{REL}= - 0.04 kJ/mol

MIN23

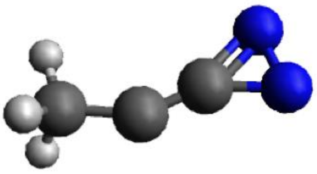

→ TS42 → [1]c-CH_2CC(N)N + H E_{REL}= - 30 kJ/mol

E_{REL}= - 206 kJ/mol

MIN17

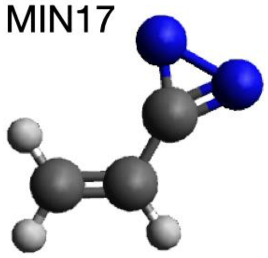

CH+

→ CH_2H + [1]c-C(N)N E_{REL}= - 2 kJ/mol

→ [1]c-CH_2CC(N)N + H E_{REL}= - 30 kJ/mol

E_{REL}= - 335 kJ/mol

MIN22

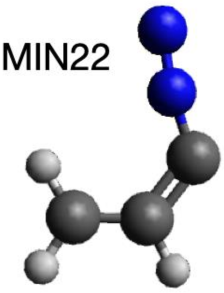

→ TS41 → CH_2CCH + [1]N_2 E_{REL}= - 516 kJ/mol

E_{REL}= - 343 kJ/mol

Table S2: Reaction Enthalpies and Barrier Heights, reported in kJ/mol, evaluated at the CCSD(T)/aug-cc-pVTZ//B3LYP/aug-cc-pVTZ level of theory for the reaction $N(^2D) + CH_2CHCN$.

process	ΔH^0_0	Barrier height	process	ΔH^0_0	Barrier height
$N(^2D)+CH_2CHCN \rightarrow$ MIN1	-414		MIN1 → ^1c-$CH_2(N)CCN+H$	191	215
MIN1 → MIN3	47	189	MIN1 → ^1c-CH(N)CHCN+H	178	193
MIN1 → MIN4	81	260	MIN3 → ^1c-CH(NH)CH+CN	350	
MIN1 → MIN12	-85	130	MIN3 → ^1c-CH(NH)CCN+H	269	275
MIN1 → MIN5	82	250	MIN3 → ^1c-C(NH)CHCN+H	251	
MIN3 → MIN2	-155	75	MIN3 → ^1c-CH(N)CHCN+H	131	148
MIN3 → MIN7	174	321	MIN4 → ^1c-$CH_2(NCN)C+H$	293	
MIN7 → MIN8	-30	173	MIN4 → ^1c-CH(NCN)CH+H	263	272
MIN5 → MIN14	41	104	MIN4 → ^1c-$CH_2(N)CH+CN$	180	
MIN5 → MIN6	7	181	MIN5 → ^1c-CH(N)CCHN+H	309	
MIN12 → MIN13	158	339	MIN5 → ^1c-$CH_2(N)CCN+H$	109	128
MIN12 → MIN20	206	338	MIN5 → c-$CH_2(N)C+^1HCN$	65	110
MIN14 → MIN20	-2	62	MIN6 → ^1c-CH(N)CCNH+H	257	271
MIN8 → MIN16	-153	39	MIN6 → ^1c-$CH_2(N)C+^1HNC$	115	147
MIN6 → MIN11	-25	132	MIN6 → ^1c-$CH_2(N)CCN+H$	102	128
MIN11 → MIN20	57	216	MIN7 → ^1c-CH(N)CCHN+H	170	
MIN13 → MIN19	-109	191	MIN7 → ^1c-CH(NH)CCN+H	95	
MIN2 → MIN21	-1	38	MIN7 → c-CH(NH)C+^{1}HCN	43	131
MIN21 → MIN9	3	67	MIN8 → ^1c-CH(N)CCNH+H	135	167
MIN9 → MIN10	115	192	MIN8 → c-CH(NH)C+^{1}HCN	130	
MIN10 → MIN15	111	207	MIN8 → ^1c-CH(NH)CCN+H	125	152
MIN15 → MIN22	-49	146	MIN14 → ^1c-$CH_2NC(N)C+H$	244	
MIN22 → MIN17	10	190	MIN14 → H_2CN+^1c-C(N)CH	172	
MIN17 → MIN23	127	321	MIN2 → HCNH+^{1}HCCN	468	
MIN23 → MIN18	-139	1	MIN2 → ^{3}CHNHCCN+H	431	
MIN22 → MIN18	-2	240	MIN2 → HCNH+^{3}HCCN	418	
MIN2 → ^{1}HNCCHCN+H	203	226	MIN2 → ^{3}CHNCHCN+H	361	
MIN21 → HCNH+^{1}HCCN	469				

process	ΔH^0_0	Barrier height	process	ΔH^0_0	Barrier height
MIN1 → ^1c-$CH_2(N)CH$+CN	261		MIN21 → ^{3}CHNHCCN+H	432	
MIN21 → HCNH+^{3}HCCN	419		MIN16 → ^{3}NCHCCNH+H	364	
MIN21 → ^{3}NCHCHCN+H	369		MIN16 → ^{3}NHCHCCN-i+H	286	
MIN21 → ^{1}NHCCHCN+H	204	239	MIN16 → ^{1}NHCCCNH+H	159	
MIN12 → $^3CH_2NCCN$+H	429		MIN20→ $^3CH_2NCCN$+H	223	
MIN12 → ^{3}CHNCHCN+H	412		MIN20 → $^1CH_2NCCN$+H	99	
MIN12 → CH_2N+^{1}HCCN	391		MIN20 → CH_2NC+^{1}HCN	-89	20
MIN12 → CH_2N+^{3}HCCN	342		MIN13→ $^3CH_2NCCN$+H	271	
MIN12 → $^1CH_2NCCN$+H	305	304	MIN13→ $^1CH_2NH$+CCN	235	
MIN12 → ^{1}CHNCHCN+H	299	299	MIN13→ $^1CH_2NCCN$+H	147	
MIN12 → ^{1}CNCHCN+H_2	134	349	MIN19→ ^{3}NCCN+CH_3	445	
MIN11 → ^{1} CH_2N+^{1}CCNH	343		MIN19→ $^3CH_2NCCN$+H	380	
MIN11 → ^{1} CH_2N+^{3}CCNH	339		MIN19→ $^1CH_2NCCN$+H	256	
MIN11 → ^{1}CHNCCNH+H	289		MIN19→ ^{1}NCCN+CH_3	30	
MIN11 → $^3CH_2NCCN$+H	280		MIN18→ ^{1}CCNN+CH_3	282	
MIN11 → $^1CH_2NCCN$+H	156		MIN18→ $^1CH_2CCNN$+H	249	
MIN9 → ^{1}HCCN+HCNH	466		MIN18→ CH_3CC+N_2	10	53
MIN9 → ^{3}NHCHCCN-i+H	430		MIN10 → ^1c-$C_3H_2N_2$+H	404	
MIN9 → ^{3}HCCN+HCNH	416		MIN15 → ^1c-$C_3H_2N_2$+H	293	312
MIN9 → ^{3}NCHCHCN+H	366		MIN23 → ^1c-$CH_2CC(N)N$+H	176	183
MIN9 → ^{3}NHCCHCN+H	201	224	MIN17 → CH_2CH+ ^1c-C(N)N	333	
MIN17 → ^1c-$CH_2CC(N)N$+H	305		MIN22 → CH_2CCH+N_2	-173	47

In the following pages are reported the structures of all the stationary points identified at the B3LYP/aug-cc-pVTZ level of theory for the N(^{2}D) + CH_2CHCN reaction. In details, the geometries are reported in the form of the z-matrix where the first column indicates the bond distances (in Å), the second column reports the bond angles and the third column reports the dihedral angles (both expressed in degrees °).

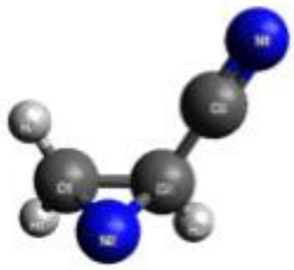

C						
C	1	1.48413				
C	2	1.43796	1	121.51173		
N	2	2.58852	1	121.46200	3	359.63806
N	1	1.44099	2	60.63778	3	255.31827
H	1	1.08512	2	118.39739	3	149.13959
H	1	1.08514	2	119.58098	3	359.80186
H	2	1.08454	1	118.56514	3	207.49882

MIN1

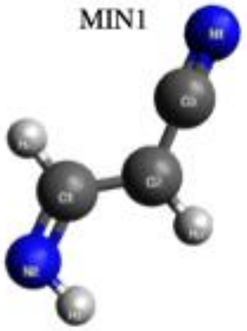

C						
C	1	1.42041				
C	2	1.39590	1	122.60438		
N	2	2.55624	1	122.66544	3	359.99851
N	1	1.29497	2	125.69643	3	179.97771
H	5	1.02254	1	111.14508	2	0.00888
H	1	1.08781	2	117.14578	3	359.97537
H	2	1.08330	1	119.84721	3	180.00045

MIN2

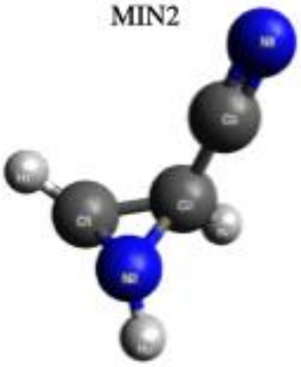

C						
C	1	1.47050				
C	2	1.44290	1	123.08557		
N	2	2.59317	1	123.14645	3	358.81764
N	1	1.38732	2	62.95597	3	259.30795
H	1	1.08061	2	133.23342	3	15.82347
H	2	1.08423	1	117.10265	3	208.37355
H	5	1.01588	1	118.01344	2	255.80009

MIN3

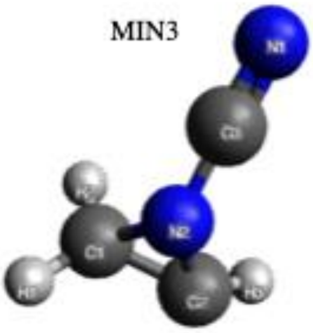

C						
C	1	1.46027				
C	2	2.43280	1	75.64853		
N	3	1.15590	2	154.55843	1	232.01177
N	3	1.33829	2	28.12325	1	58.07189
H	1	1.08143	2	120.62603	3	235.71972
H	1	1.08475	2	119.16431	3	79.20453
H	2	1.08048	1	130.93427	3	270.95231

MIN4

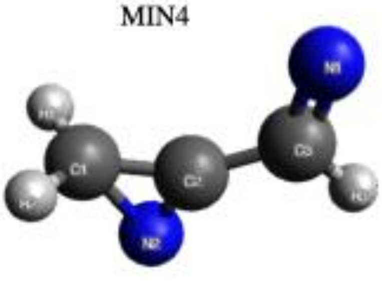

C						
C	1	1.44353				
C	2	1.46116	1	152.60936		
N	3	1.24853	2	123.58389	1	0.11612
N	2	1.25202	1	69.73476	3	180.18661
H	1	1.08268	2	120.42708	3	80.24268
H	1	1.08266	2	120.46259	3	279.43136
H	3	1.09744	2	116.00579	1	180.09011

MIN5

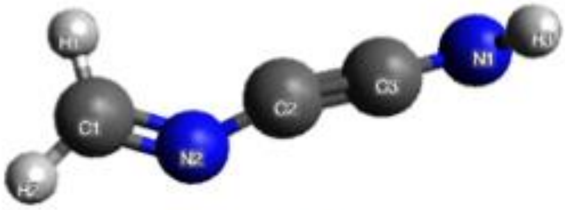

MIN11

C						
C	1	2.36208				
C	2	1.25507	1	173.30954		
N	3	1.24944	2	171.70926	1	289.00692
N	1	1.27356	2	22.77085	3	191.29783
H	1	1.08842	2	99.60938	3	10.86812
H	1	1.08233	2	141.33908	3	190.61765
H	4	1.01528	3	117.58679	2	153.47604

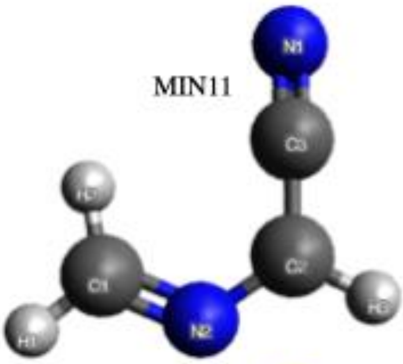

MIN12

C						
C	1	2.30439				
C	2	1.41411	1	95.80372		
N	2	2.57182	1	95.37976	3	0.00021
N	1	1.30006	2	29.86353	3	179.98919
H	1	1.08253	2	147.92001	3	179.97368
H	1	1.09094	2	93.59184	3	359.98808
H	2	1.08104	1	146.58686	3	179.99895

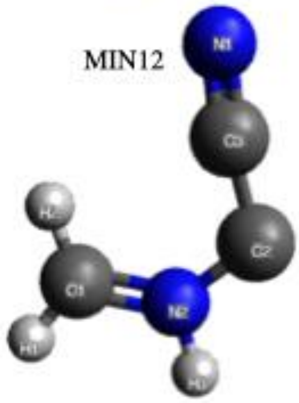

MIN13

C						
C	1	2.45376				
C	2	1.40554	1	89.93147		
N	3	1.16284	2	172.21330	1	180.10975
N	2	1.34553	1	24.56896	3	180.04153
H	1	1.07759	2	143.11272	3	179.99361
H	1	1.07765	2	94.19155	3	359.99155
H	5	1.01346	2	113.99596	1	180.01510

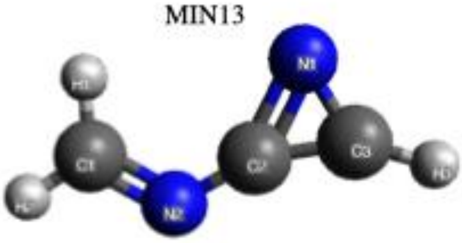

MIN14

C						
C	1	2.27078				
C	2	1.35188	1	170.47510		
N	3	1.30452	2	67.72048	1	0.14630
N	2	1.28411	1	30.43000	3	180.15123
H	1	1.08621	2	93.20937	3	359.79481
H	1	1.07967	2	147.02558	3	179.88025
H	3	1.08312	2	155.27275	1	180.15836

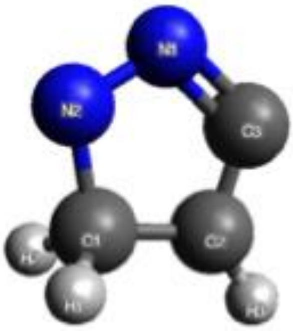

MIN15

C						
C	1	1.48967				
H	1	1.09391	2	114.39297		
H	1	1.09388	2	114.30550	3	235.86326
C	2	1.32990	1	102.67130	3	242.00522
H	2	1.07530	1	127.55533	3	62.03188
N	5	1.40607	2	114.43586	1	0.01521
N	7	1.26059	5	108.43173	2	359.98853

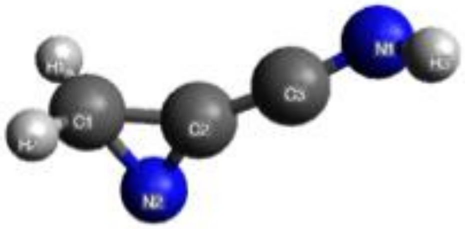

MIN6

C						
C	1	1.46101				
C	2	1.32285	1	150.25975		
N	3	1.21436	2	169.46774	1	275.34060
N	2	1.32157	1	64.56729	3	174.43277
H	1	1.08532	2	119.09587	3	81.85062
H	1	1.08537	2	119.61357	3	288.51215
H	4	1.01870	3	120.71285	2	170.28279

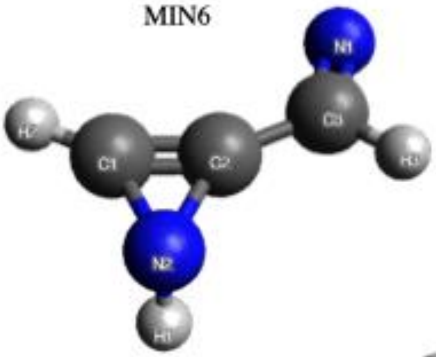

MIN7

C						
C	1	1.27712				
C	2	1.43863	1	157.39000		
N	3	1.25594	2	124.53457	1	343.43125
N	1	1.49596	2	66.10627	3	193.05591
H	5	1.02325	1	108.14793	2	260.98705
H	1	1.07247	2	155.42927	3	2.58430
H	3	1.09709	2	116.45091	1	163.66273

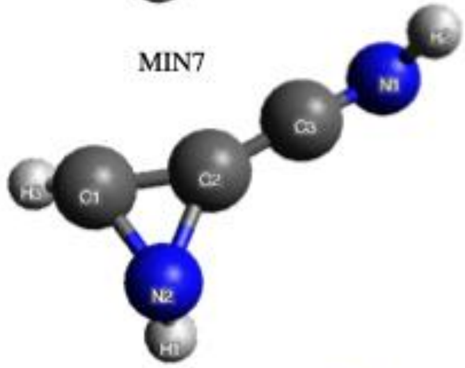

MIN8

C						
C	1	1.39163				
C	2	1.29225	1	158.99765		
N	3	1.22698	2	172.34883	1	269.36726
N	1	1.43095	2	63.35740	3	183.77630
H	5	1.02318	1	113.52443	2	257.86099
H	4	1.02031	3	116.11568	2	179.88952
H	1	1.08069	2	134.69101	3	63.31972

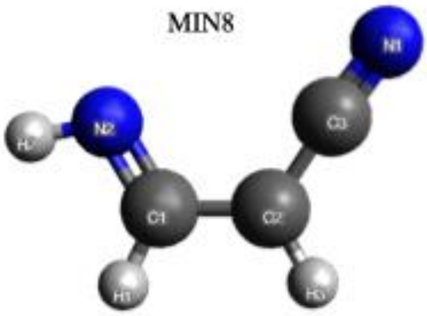

MIN9

C						
C	1	1.41955				
H	1	1.09147	2	115.45191		
H	1	1.91287	2	151.37042	3	179.98219
C	2	1.39875	1	123.59584	3	180.05733
H	2	1.08151	1	119.49415	3	0.06201
N	2	2.55664	1	124.85079	3	180.05924
N	4	1.01867	1	39.16777	2	0.01038

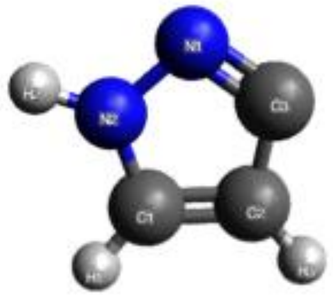

MIN10

C						
C	1	1.38148				
H	1	1.07710	2	131.12999		
H	1	2.12997	2	128.87948	3	179.85213
C	2	1.40673	1	102.34859	3	180.13433
H	2	1.07326	1	128.17504	3	359.98703
N	5	1.29232	2	114.92393	1	359.99341
N	4	1.00445	1	29.91918	2	0.05418

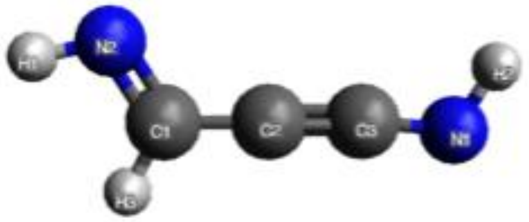

MIN16

C						
C	1	1.39984				
C	2	1.24724	1	179.97438		
N	3	1.25427	2	174.08611	1	91.66048
N	1	1.29294	2	121.52440	3	88.34434
H	5	1.02075	1	109.98720	2	180.00454
H	4	1.01977	3	114.07047	2	179.88126
H	1	1.09314	2	116.40335	3	268.34743

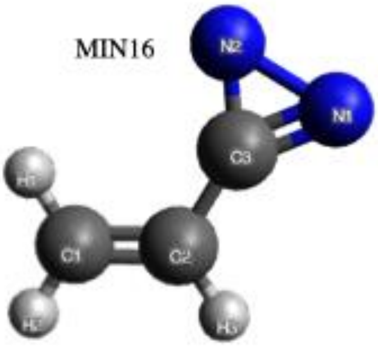

MIN17

C						
C	1	1.33130				
H	1	1.08194	2	121.11034		
H	1	1.08079	2	121.37253	3	180.05970
C	2	1.44224	1	120.97488	3	0.18433
H	2	1.08201	1	122.33089	3	180.00492
N	5	1.29807	2	143.56535	1	179.49120
N	5	1.31521	2	143.65959	1	359.58295

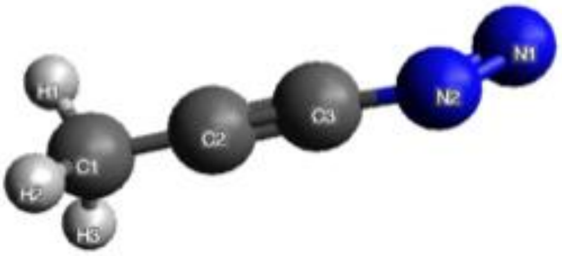

MIN18

C						
C	1	1.45118				
H	1	1.09110	2	110.87838		
H	1	1.09092	2	111.38676	3	239.14591
C	3	3.19882	1	51.18506	2	0.96206
H	1	1.09156	2	110.24976	3	119.16370
N	5	2.31047	2	168.06293	1	3.77317
N	7	1.18148	5	26.07366	2	180.67675

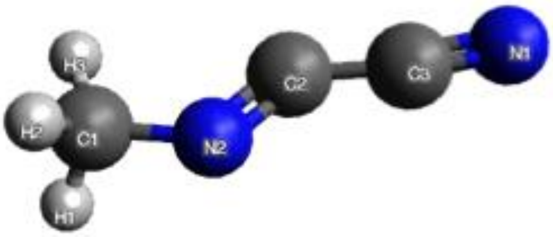

MIN19

C						
C	1	2.40862				
H	1	1.08944	2	119.12424		
H	1	1.08945	2	119.20949	3	135.18202
C	2	1.38366	1	166.32438	3	292.53123
H	1	1.09028	2	88.85592	3	247.60004
N	5	1.16092	2	170.49350	1	179.82667
N	2	1.22236	1	28.14013	3	292.34580

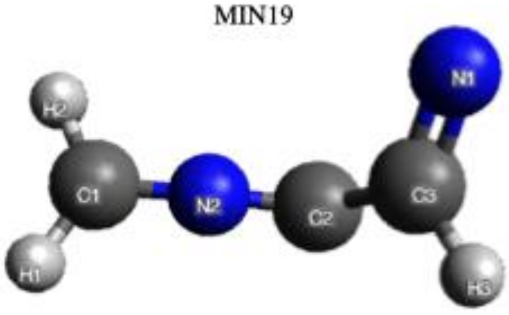

MIN20

C						
C	1	2.48000				
C	2	1.46976	1	130.41495		
N	3	1.24905	2	124.38819	1	287.50481
N	3	2.40700	1	8.02101	2	359.03458
H	1	1.08051	2	119.71390	3	266.46191
H	1	1.08054	2	119.17330	3	89.75058
H	3	1.09732	2	114.79461	1	113.34406

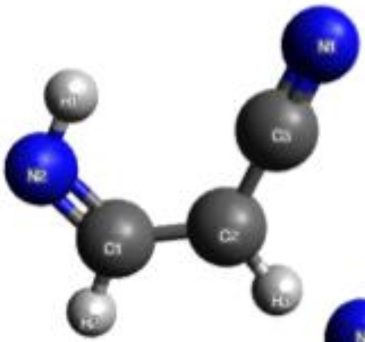

MIN21

C						
C	1	1.42257				
C	2	1.39913	1	122.70734		
N	2	2.55814	1	122.43735	3	359.99999
N	1	1.29476	2	127.46027	3	359.99982
H	5	1.02324	1	111.37344	2	0.00024
H	1	1.08775	2	115.63582	3	180.00095
H	2	1.08173	1	119.89615	3	179.99900

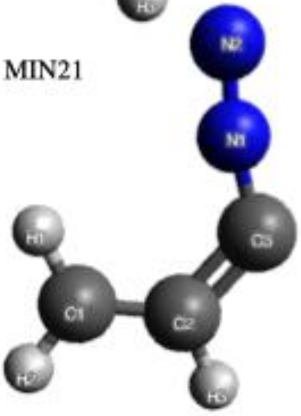

MIN22

C						
C	1	1.35702				
H	1	1.08223	2	121.80386		
H	1	1.08130	2	120.96847	3	180.03475
C	2	1.41697	1	126.02936	3	359.98788
H	2	1.08291	1	118.53757	3	179.99378
N	5	1.27769	2	123.78169	1	359.92965
N	7	1.14462	5	170.18439	2	180.56692

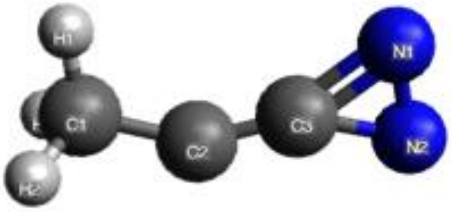

MIN23

C						
C	1	1.46096				
H	1	1.09658	2	111.13729		
H	1	1.09142	2	111.26348	3	240.01327
C	2	1.26262	1	150.45844	3	0.06913
H	1	1.09143	2	111.25629	3	119.97895
N	5	1.46942	2	156.24391	1	359.96484
N	7	1.23808	5	62.66843	2	180.02067

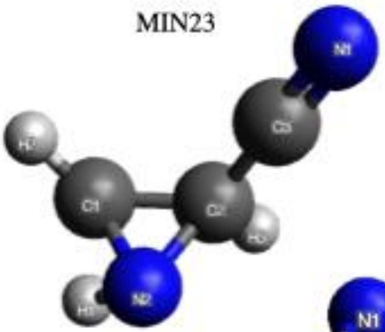

TS1

C						
C	1	1.46276				
C	2	1.43954	1	118.68865		
N	2	2.58968	1	119.15000	3	358.98415
N	1	1.45268	2	62.17248	3	259.63693
H	5	1.23186	1	56.60096	2	242.85982
H	1	1.08170	2	135.04126	3	16.15986
H	2	1.08283	1	121.07643	3	208.60482

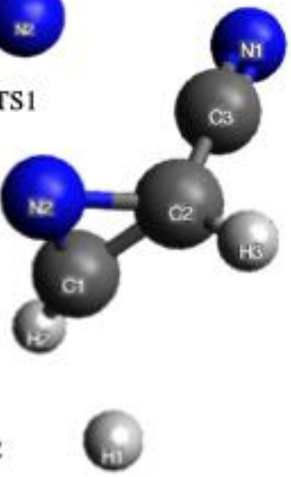

TS2

C						
C	1	1.46022				
C	2	1.43765	1	121.53232		
N	2	2.58880	1	121.73489	3	359.46185
N	1	1.25345	2	68.88325	3	259.50736
H	1	2.04931	2	99.55583	3	153.05607
H	1	1.07926	2	149.03658	3	60.99933
H	2	1.08162	1	120.02737	3	201.02992

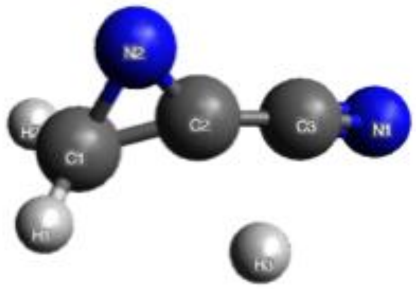

TS3

C					
C	1	1.45752			
C	2	1.41012	1	149.72448	
N	2	2.56159	1	148.41478	3	0.39612
N	2	1.26824	1	67.52599	3	155.53253
H	1	1.08194	2	119.22810	3	102.06481
H	1	1.08285	2	120.44464	3	303.74511
H	2	1.99977	1	97.71502	3	260.01139

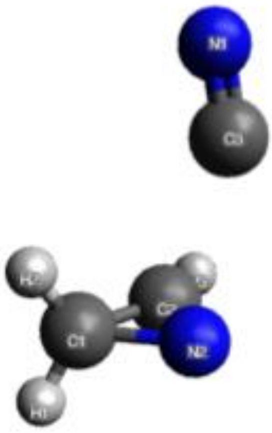

TS4

C						
C	1	1.44385				
C	2	2.54805	1	101.04628		
N	3	1.16121	2	146.99599	1	287.00614
N	2	1.26407	1	68.78264	3	74.11216
H	1	1.08370	2	120.52447	3	188.07353
H	1	1.08238	2	120.21575	3	27.85906
H	2	1.07941	1	152.19705	3	248.97503

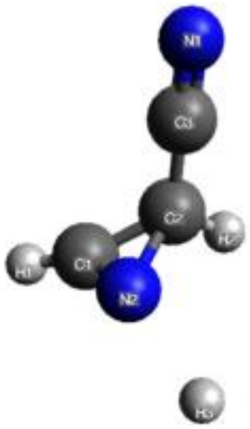

TS5

C						
C	1	1.45381				
C	2	1.43836	1	121.63602		
N	2	2.58924	1	121.89536	3	359.45388
N	1	1.25146	2	69.37188	3	258.84856
H	1	1.07885	2	149.52670	3	80.90902
H	2	1.08330	1	120.26102	3	200.16799
H	5	1.89639	1	121.56928	2	263.67664

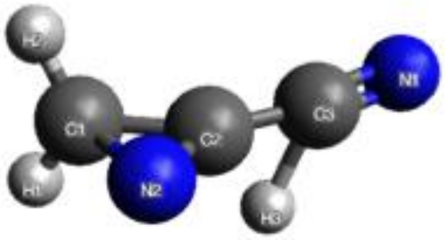

C						
C	1	1.45264				
C	2	1.40279	1	149.98658		
N	3	1.18206	2	172.37115	1	68.84424
N	2	1.29753	1	66.49941	3	173.16826
H	1	1.08410	2	119.99255	3	85.06949
H	1	1.08436	2	119.35918	3	289.42649
H	3	1.38151	2	64.85830	1	265.96302

TS6

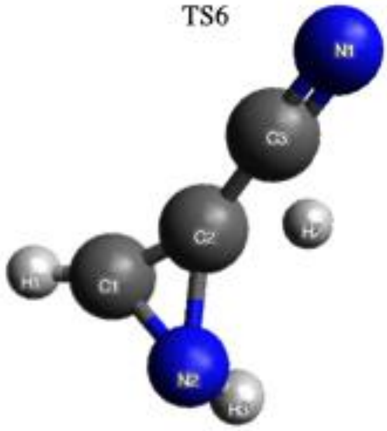

C						
C	1	1.28536				
C	2	1.38317	1	155.85949		
N	2	2.53846	1	156.38711	3	359.97513
N	1	1.45576	2	68.54067	3	212.74490
H	1	1.07442	2	152.66578	3	25.56146
H	2	2.32202	1	101.20654	3	242.10598
H	5	1.02314	1	109.56105	2	262.46439

TS7

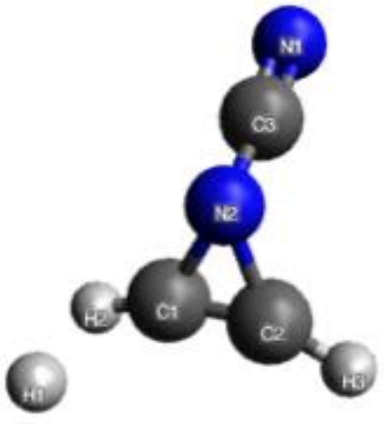

C						
C	1	1.27315				
C	2	2.41900	1	75.58593		
N	3	1.15530	2	150.20026	1	241.68894
N	3	1.36000	2	34.73613	1	66.72238
H	1	2.25479	2	107.93891	3	237.63250
H	1	1.07051	2	157.89697	3	132.54889
H	2	1.07138	1	158.35075	3	206.63855

TS8

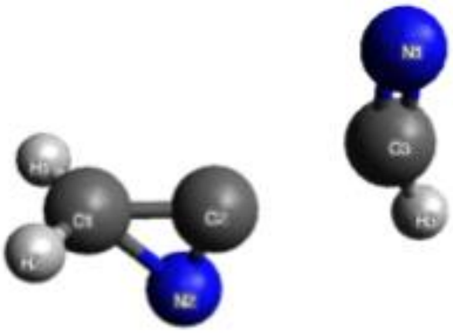

C						
C	1	1.46125				
C	2	2.14731	1	155.54178		
N	3	1.16659	2	114.68134	1	359.99929
N	2	1.22158	1	74.17787	3	179.98862
H	1	1.08031	2	120.32575	3	83.31084
H	1	1.08031	2	120.32580	3	276.71198
H	3	1.07204	2	91.05857	1	179.99860

TS9

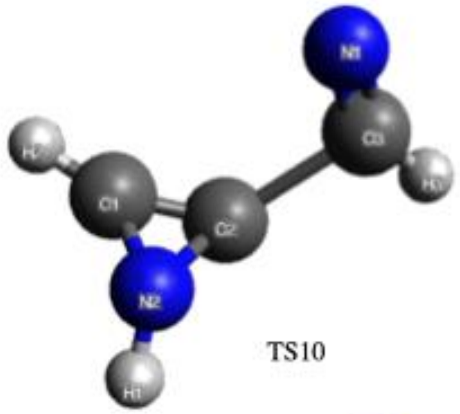

TS10

C						
C	1	1.34081				
C	2	1.94771	1	129.85062		
N	3	1.17623	2	107.31773	1	12.74259
N	1	1.37792	2	66.71955	3	252.34082
H	5	1.01872	1	124.76136	2	256.38308
H	1	1.07411	2	154.57844	3	61.71001
H	3	1.07261	2	104.29773	1	192.02353

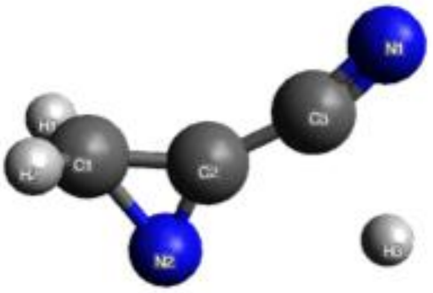

TS11

C						
C	1	1.44979				
C	2	1.41576	1	149.64418		
N	3	1.16247	2	161.98045	1	0.00000
N	2	1.25127	1	69.10094	3	180.00000
H	1	1.08242	2	120.01512	3	79.81988
H	1	1.08242	2	120.01509	3	280.18011
H	3	1.93581	2	93.84601	1	179.99997

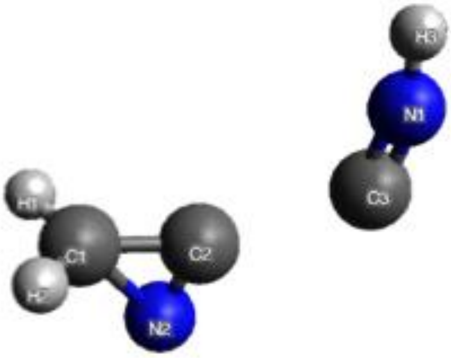

C						
C	1	1.48573				
C	2	2.21634	1	159.13062		
N	3	1.17133	2	121.85600	1	359.86579
N	2	1.22132	1	71.41010	3	179.95141
H	1	1.08103	2	120.26250	3	82.05618
H	1	1.08103	2	120.26349	3	278.03659
H	4	0.99790	3	165.04315	2	359.99535

TS12

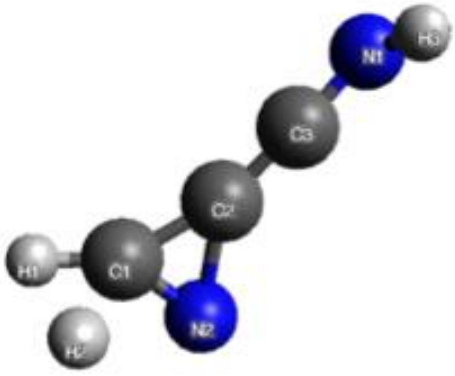

C						
C	1	1.40276				
C	2	1.28113	1	163.18373		
N	3	1.23334	2	171.24238	1	257.22697
N	1	1.26955	2	68.46899	3	193.61019
H	1	1.07948	2	150.32853	3	29.42746
H	1	2.17130	2	108.69187	3	290.61866
H	4	1.02076	3	114.74527	2	178.90679

TS13

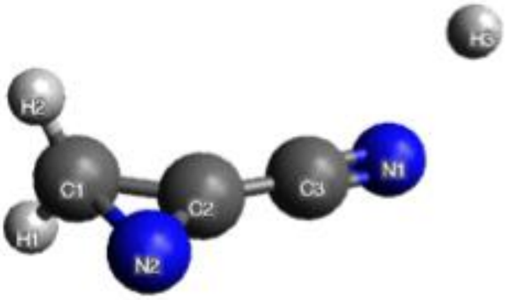

TS14

C						
C	1	1.45073				
C	2	1.39786	1	150.34979		
N	2	2.55545	1	149.05574	3	0.82544
N	2	1.25818	1	68.51310	3	180.89182
H	1	1.08261	2	119.91891	3	78.62016
H	1	1.08259	2	119.86236	3	279.75427
H	4	1.88315	3	118.73902	2	72.44304

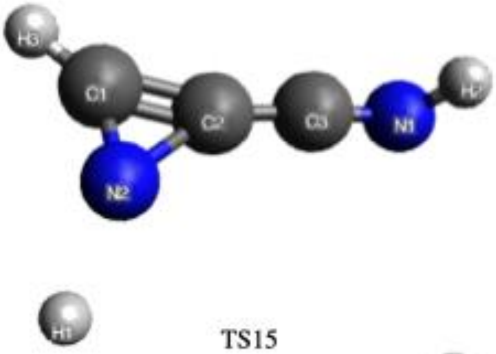

TS15

C						
C	1	1.39090				
C	2	1.28095	1	164.47150		
N	3	1.23275	2	171.62251	1	259.92390
N	1	1.26633	2	70.43894	3	191.35383
H	5	2.05016	1	117.83646	2	254.12354
H	4	1.02058	3	114.97487	2	179.37175
H	1	1.08083	2	149.87003	3	13.70150

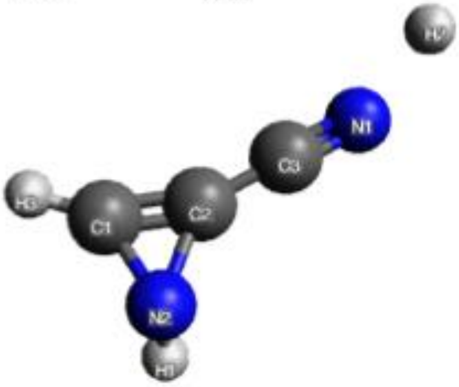

TS16

C						
C	1	1.28199				
C	2	1.37209	1	158.48733		
N	2	2.53442	1	159.01291	3	359.73893
N	1	1.47123	2	67.90895	3	186.81920
H	5	1.02424	1	108.65195	2	261.54389
H	4	1.88837	3	117.17319	2	255.61987
H	1	1.07361	2	153.32515	3	0.97955

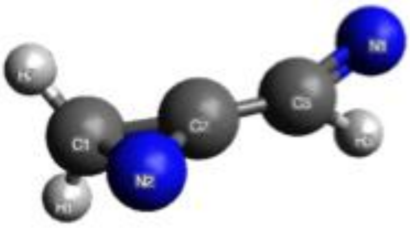

TS17

C						
C	1	1.45658				
C	2	1.39776	1	148.25793		
N	3	1.24406	2	154.42513	1	104.07383
N	2	1.27528	1	67.12012	3	198.45855
H	1	1.08412	2	120.47607	3	61.33968
H	1	1.08372	2	119.08077	3	264.63823
H	3	1.22233	2	142.02245	1	291.24233

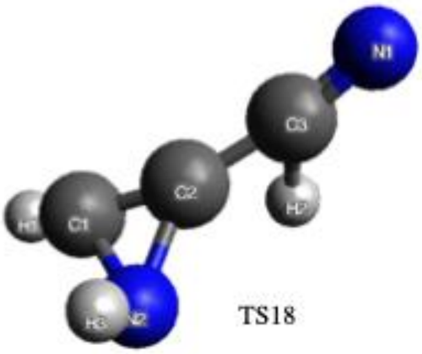

TS18

C						
C	1	1.32676				
C	2	1.39661	1	158.68614		
N	3	1.18827	2	167.47670	1	93.82943
N	1	1.46939	2	64.12674	3	167.63602
H	1	1.07285	2	149.73092	3	29.60414
H	3	1.30933	2	68.51834	1	274.07496
H	5	1.02243	1	111.00492	2	99.35570

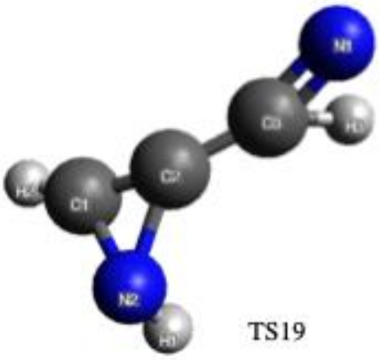

TS19

C						
C	1	1.30300				
C	2	1.37667	1	154.70432		
N	3	1.24947	2	154.67105	1	108.09371
N	1	1.46070	2	67.88197	3	139.37126
H	5	1.02865	1	108.70467	2	259.31164
H	1	1.07294	2	151.91087	3	336.11630
H	3	1.22070	2	141.13333	1	294.31889

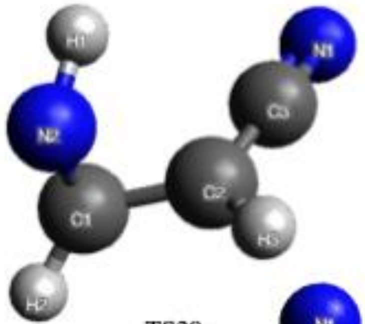

TS20

C						
C	1	1.45547				
C	2	1.40968	1	121.99748		
N	2	2.56606	1	121.74988	3	0.55518
N	1	1.33505	2	86.12000	3	260.36917
H	5	1.01928	1	115.34924	2	66.36000
H	1	1.08084	2	130.53889	3	126.77270
H	2	1.08038	1	120.05297	3	177.20408

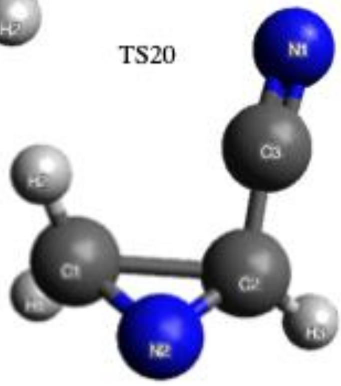

TS21

C						
C	1	1.90526				
C	2	1.43727	1	95.71208		
N	3	1.15533	2	174.50178	1	170.60227
N	2	1.34918	1	46.61021	3	138.83072
H	1	1.08300	2	114.64181	3	115.76314
H	1	1.08767	2	112.78191	3	335.36618
H	2	1.09095	1	140.62180	3	221.42865

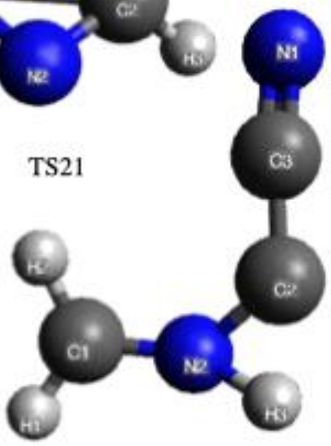

TS22

C						
C	1	2.43963				
C	2	1.39473	1	104.85923		
N	3	1.16281	2	174.10044	1	181.32540
N	1	1.31021	2	24.82317	3	158.10323
H	1	1.07904	2	143.10147	3	170.83803
H	1	1.08522	2	95.70608	3	344.07512
H	5	1.20645	1	143.91192	2	101.38188

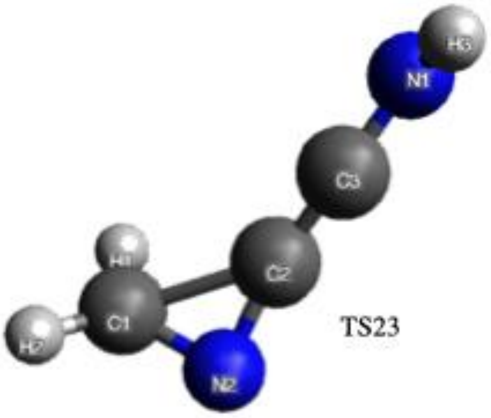

TS23

C						
C	1	1.84895				
C	2	1.27267	1	144.69776		
N	3	1.24142	2	167.06794	1	269.86722
N	2	1.32754	1	47.14302	3	153.82567
H	1	1.08480	2	93.11190	3	70.71286
H	1	1.08996	2	137.58665	3	292.17656
H	4	1.01470	3	119.11289	2	178.79926

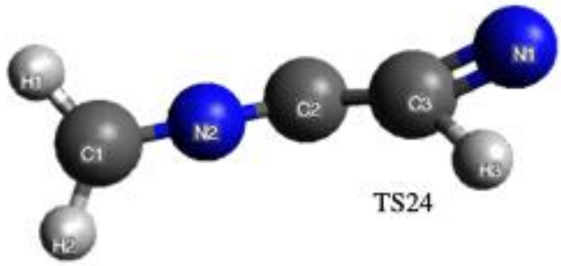

TS24

C						
C	1	2.48833				
C	2	1.35842	1	142.07863		
N	3	1.25799	2	156.51208	1	233.67585
N	3	2.42123	1	5.51098	2	359.44519
H	1	1.08350	2	119.34866	3	105.95130
H	1	1.08390	2	120.67692	3	283.18207
H	3	1.20446	2	138.10287	1	52.19052

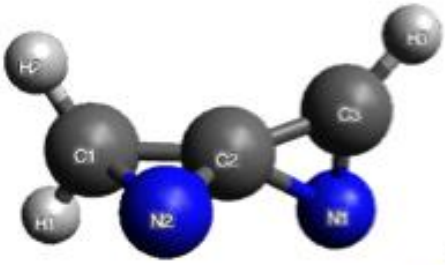

TS25

C						
C	1	1.44423				
C	2	1.43748	1	151.57235		
N	3	1.24464	2	77.02147	1	252.70514
N	2	1.33297	1	66.41235	3	161.16377
H	1	1.08448	2	120.41796	3	99.31632
H	1	1.08518	2	120.74992	3	300.70875
H	3	1.08581	2	144.09187	1	73.81877

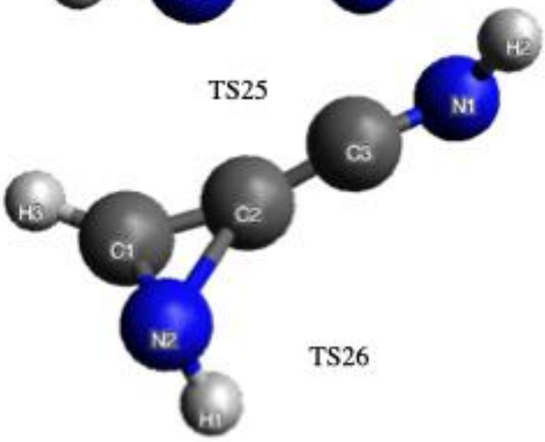

TS26

C						
C	1	1.33717				
C	2	1.29521	1	163.97417		
N	3	1.23026	2	164.58603	1	213.31493
N	1	1.38040	2	84.16921	3	265.97716
H	5	1.01711	1	111.54274	2	281.16629
H	4	1.01798	3	118.51204	2	177.89210
H	1	1.07977	2	139.46696	3	59.49284

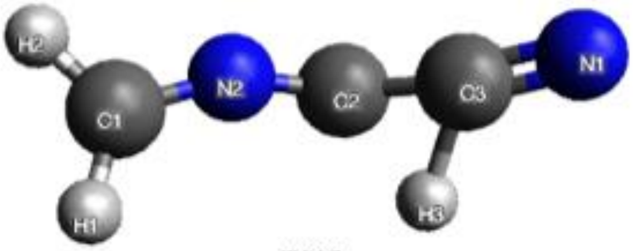

TS27

C						
C	1	2.47375				
C	2	1.37226	1	133.91971		
N	3	1.20568	2	166.64930	1	223.14567
N	2	1.25351	1	10.46144	3	274.05339
H	1	1.08735	2	111.06302	3	283.37849
H	1	1.08329	2	129.58539	3	106.88124
H	3	1.33028	2	65.87033	1	94.22476

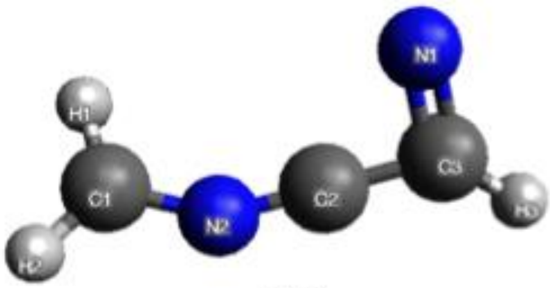

TS28

C					
C	1	2.43962			
C	2	1.37496	1	164.65890		
N	3	1.27319	2	90.11722	1	30.64017
N	2	1.21191	1	14.88483	3	183.74737
H	1	1.08302	2	106.77224	3	9.81281
H	1	1.07878	2	132.22597	3	192.24423
H	3	1.09330	2	134.89548	1	215.92282

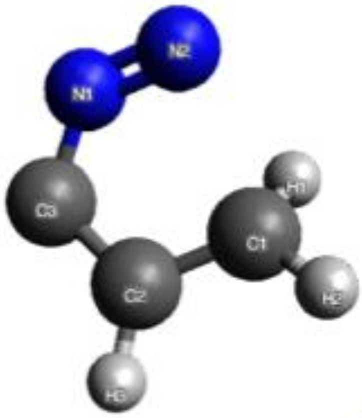

TS29

C						
C	1	1.41068				
H	1	1.08736	2	119.47961		
H	1	1.08395	2	119.66619	3	212.30124
C	2	1.37492	1	115.76960	3	304.75880
H	2	1.08123	1	121.98380	3	128.82835
N	5	1.31623	2	110.96198	1	348.88146
N	7	1.18935	5	129.14424	2	357.98211

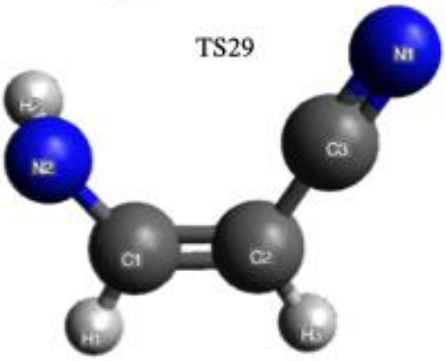

TS30

C						
C	1	1.34283				
H	1	1.09120	2	118.57216		
H	1	2.00656	2	124.19343	3	208.24513
C	2	1.41917	1	123.21496	3	180.65307
H	2	1.07988	1	119.42194	3	0.79130
N	2	2.57264	1	123.61637	3	180.40641
N	4	1.02436	1	39.25050	2	253.33191

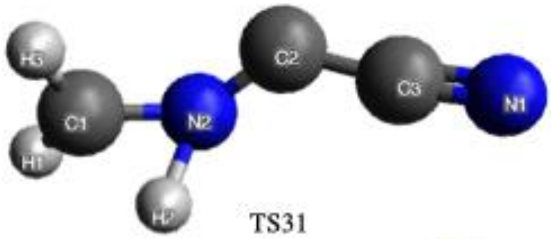

TS31

C						
C	1	2.44341				
H	1	1.07890	2	135.07751		
H	1	1.43349	2	64.96479	3	72.80257
C	2	1.37716	1	147.08661	3	278.79085
H	1	1.08436	2	98.82989	3	206.31635
N	5	1.16378	2	172.73713	1	219.64545
N	4	1.20226	1	62.53729	2	340.14257

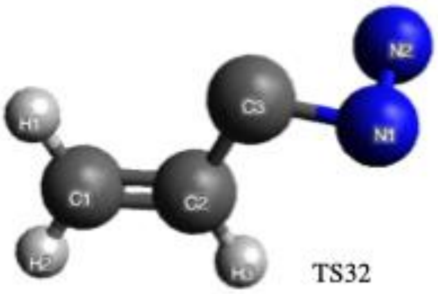

TS32

C						
C	1	1.34340				
H	1	1.08158	2	120.14207		
H	1	1.08208	2	121.75567	3	181.18934
C	2	1.43936	1	118.25113	3	4.35331
H	2	1.08849	1	120.18484	3	179.92967
N	5	1.44465	2	109.99959	1	173.98350
N	7	1.18956	5	89.40658	2	115.55562

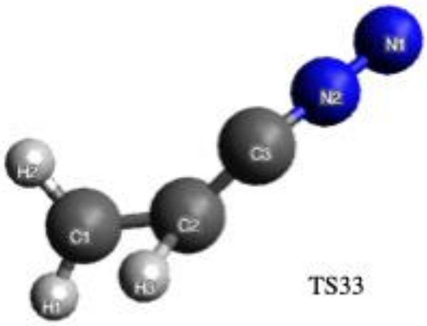

TS33

C						
C	1	1.38428				
H	1	1.08220	2	119.50724		
H	1	1.09011	2	123.34932	3	184.99898
C	2	1.32073	1	139.40174	3	185.14492
H	2	1.25570	1	63.39739	3	291.46121
N	5	2.40206	2	147.55099	1	244.60641
N	2	2.44953	1	143.89641	3	158.84284

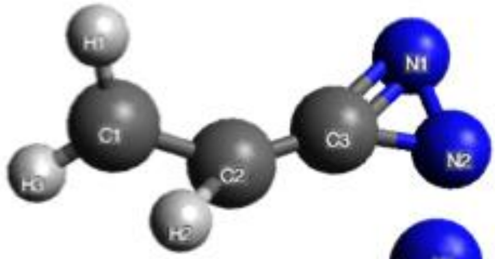

TS34

C						
C	1	1.42854				
H	1	1.08502	2	121.95262		
H	2	1.28369	1	59.13994	3	106.26151
C	2	1.29162	1	145.93799	3	336.06896
H	1	1.08078	2	118.62080	3	167.97960
N	5	1.44335	2	155.38748	1	323.44020
N	7	1.25294	5	61.95042	2	185.23931

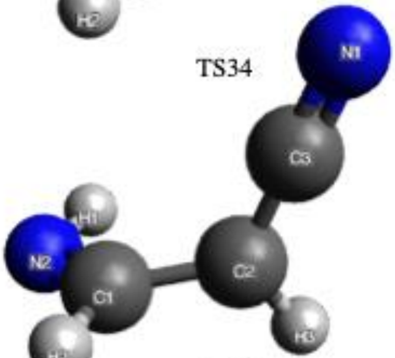

TS35

C						
C	1	1.48795				
C	2	1.37967	1	122.25549		
N	2	2.54528	1	122.04985	3	359.95716
N	1	1.26448	2	125.34692	3	91.39238
H	5	1.02208	1	111.87746	2	358.65095
H	1	1.09178	2	116.57792	3	270.26839
H	2	1.08314	1	119.17416	3	178.66512

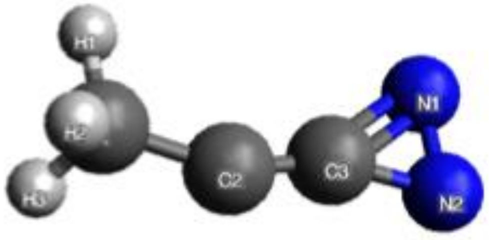

TS36

C						
C	1	1.45716				
H	1	1.09573	2	111.24704		
H	1	1.09141	2	111.13621	3	239.92627
C	2	1.24826	1	156.13001	3	0.03658
H	1	1.09141	2	111.13656	3	120.07232
N	5	1.56818	2	152.34007	1	0.00270
N	7	1.23334	5	58.46008	2	179.99929

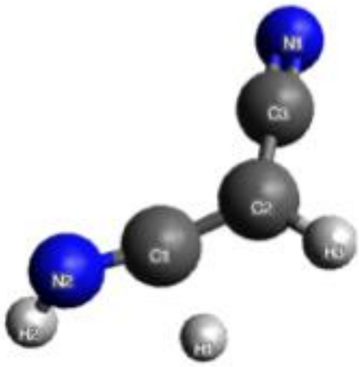

TS37

C						
C	1	1.34083				
H	1	1.94348	2	91.59020		
H	1	1.92656	2	169.93413	3	343.22737
C	2	1.41000	1	121.86816	3	99.02638
H	2	1.08162	1	118.59585	3	285.56284
N	2	2.56458	1	122.51921	3	99.14236
N	4	1.01521	1	33.07617	2	187.19601

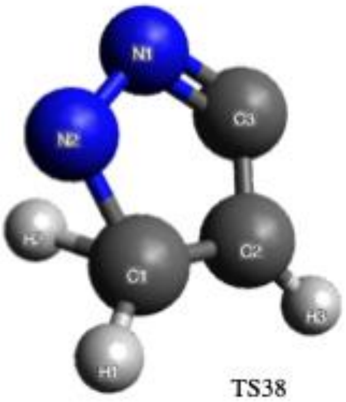

TS38

C						
C	1	1.40380				
H	1	1.07990	2	130.42058		
H	1	1.26701	2	112.81397	3	223.98888
C	2	1.38088	1	101.73654	3	196.41329
H	2	1.07421	1	128.65997	3	16.35933
N	5	1.31100	2	115.55170	1	358.44179
N	4	1.31937	1	68.56146	2	261.98088

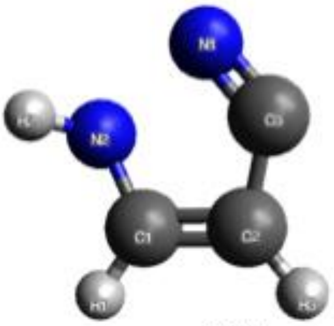

TS39

C						
C	1	1.35984				
H	1	1.08303	2	126.05470		
H	1	2.05344	2	128.21388	3	157.74062
C	2	1.42849	1	103.75392	3	184.42844
H	2	1.07372	1	128.32754	3	4.82093
N	5	1.20365	2	127.69943	1	359.23589
N	4	1.01798	1	36.08000	2	66.81549

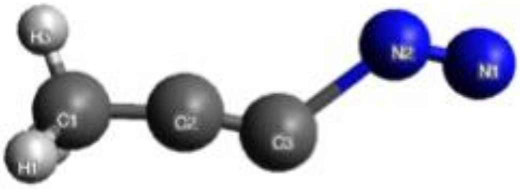

TS40

C						
C	1	1.45247				
H	1	1.09106	2	111.18715		
H	1	1.09092	2	111.23677	3	238.87522
C	2	1.22685	1	168.95885	3	56.81343
H	1	1.09301	2	110.22542	3	119.36682
N	5	2.71997	2	149.14153	1	180.10778
N	7	1.10640	5	31.48954	2	0.15527

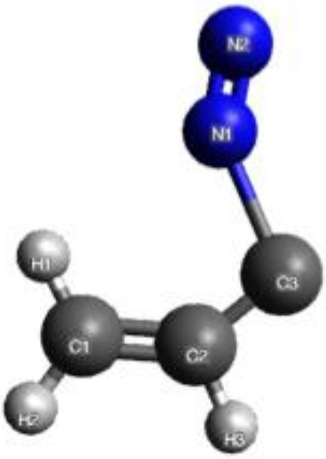

TS41

C						
C	1	1.38236				
H	1	1.08028	2	120.76898		
H	1	1.07898	2	121.09228	3	180.00000
C	2	1.36104	1	136.42409	3	0.00000
H	2	1.09598	1	118.17284	3	180.00000
N	5	1.91814	2	105.84406	1	0.00000
N	7	1.09636	5	146.70057	2	180.00000

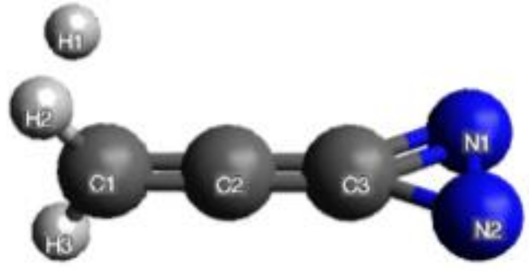

C						
C	1	1.31125				
H	1	2.36776	2	105.39080		
H	1	1.08308	2	121.39427	3	268.03658
C	3	3.92659	1	39.44688	2	0.00000
H	1	1.08308	2	121.39430	3	91.96342
N	5	1.42313	2	154.15214	1	0.00000
N	7	1.24170	5	64.09649	2	180.00000

TS42

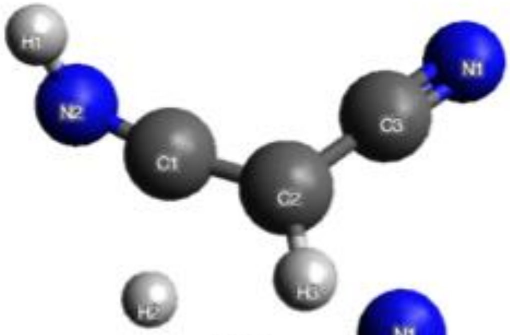

C						
C	1	1.34050				
C	2	1.41212	1	120.94722		
N	2	2.56687	1	121.54433	3	0.12896
N	1	1.21259	2	168.93651	3	323.82603
H	5	1.01769	1	118.47376	2	310.86645
H	1	1.88183	2	90.14706	3	123.19009
H	2	1.07982	1	119.72042	3	183.80550

TS43

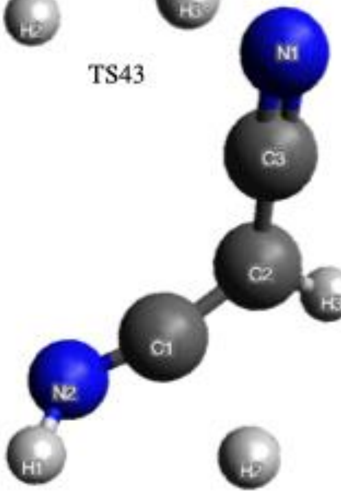

C						
C	1	1.34083				
C	2	1.41000	1	121.86837		
N	2	2.56457	1	122.51960	3	359.88388
N	1	1.21015	2	162.69606	3	86.62889
H	5	1.01521	1	119.67721	2	184.22083
H	1	1.94350	2	91.58954	3	260.97229
H	2	1.08162	1	118.59587	3	173.46428

TS45

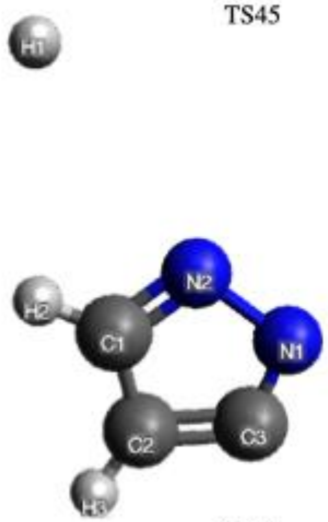

C						
C	1	1.39745				
H	1	3.86064	2	179.97438		
H	1	1.07929	2	128.64791	3	172.07082
C	2	1.46762	1	101.78561	3	7.91104
H	2	1.07645	1	130.22417	3	188.03906
N	5	1.24227	2	114.97825	1	0.00287
N	1	1.32624	2	111.85875	3	352.34426

TS44

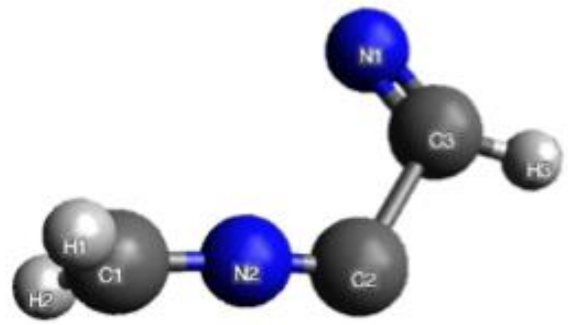

TS46

C						
C	1	2.49264				
C	2	1.82020	1	124.17355		
N	3	1.18981	2	114.11341	1	348.92780
N	3	2.66457	1	10.40854	2	359.28012
H	1	1.07971	2	119.50227	3	267.88861
H	1	1.07976	2	118.97530	3	90.22527
H	3	1.07782	2	105.38979	1	169.66961

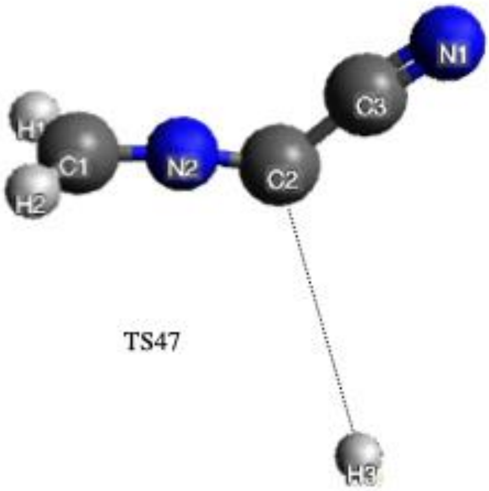

TS47

C						
C	1	2.47853				
C	2	1.39396	1	132.09059		
N	3	1.15792	2	172.72636	1	180.00000
N	3	2.35394	1	7.23860	2	0.00000
H	1	1.08137	2	119.50115	3	91.69634
H	1	1.08137	2	119.50113	3	268.30360
H	2	4.73075	1	110.49064	3	180.00000

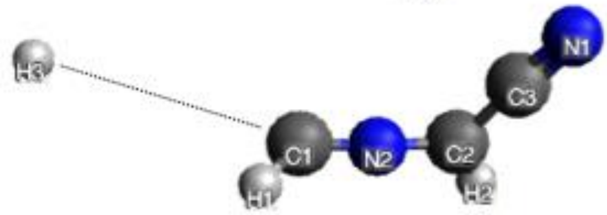

TS48

C						
C	1	2.48309				
C	2	1.41155	1	121.19787		
N	2	2.56644	1	122.03185	3	359.99455
N	3	2.35523	1	10.43597	2	349.06824
H	1	1.08464	2	129.91015	3	267.99512
H	2	1.07987	1	118.44945	3	183.94146
H	6	4.51344	1	85.50914	2	186.00696

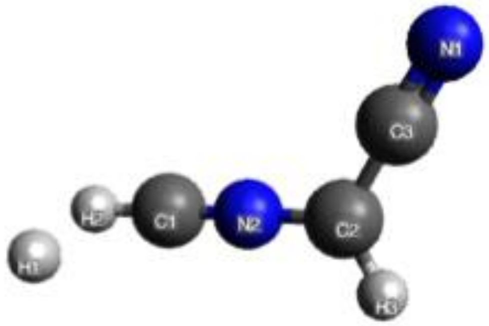

TS49

C						
C	1	2.47873				
C	2	1.40191	1	120.37993		
N	2	2.55943	1	120.97400	3	359.96768
N	3	2.35655	1	10.03183	2	358.06741
H	1	2.37232	2	106.37280	3	264.47953
H	1	1.06263	2	171.95290	3	270.73606
H	2	1.07755	1	117.58245	3	181.00797

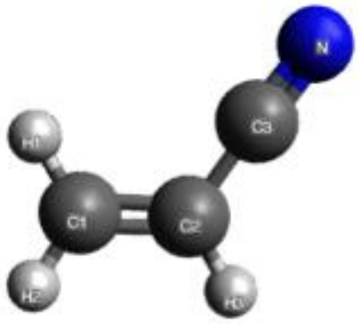

C						
C	1	1.33116				
H	1	1.08074	2	121.75956		
H	1	1.08038	2	120.57518	3	180.00000
C	2	1.42622	1	123.13455	3	0.00000
H	2	1.08274	1	121.21305	3	180.00000
N	2	2.57849	1	123.75543	3	0.00000

C_3H_3N

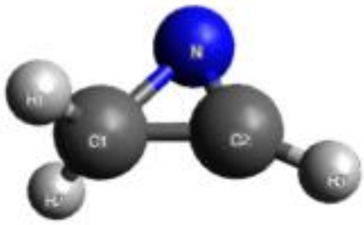

C						
C	1	1.44757				
N	2	1.24616	1	69.65658		
H	1	1.08314	2	120.63235	3	260.45363
H	1	1.08313	2	120.61501	3	99.54373
H	2	1.08061	1	151.43544	3	180.02381

^1c-$CH_2(N)CH$

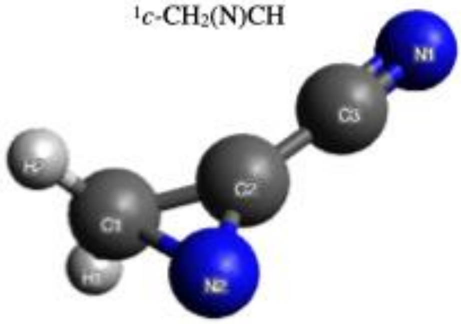

C						
C	1	1.44979				
C	2	1.40599	1	150.39673		
N	2	2.55738	1	149.05293	3	359.97233
N	2	1.25433	1	68.90462	3	180.07111
H	1	1.08243	2	119.93940	3	79.75227
H	1	1.08236	2	120.00372	3	280.19239

^1c-$CH_2(N)CCN$

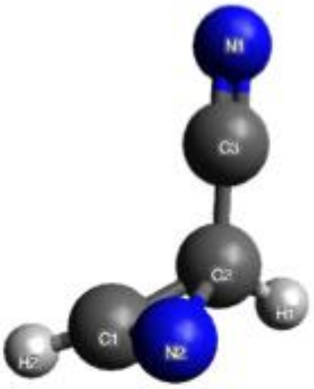

C						
C	1	1.45579				
C	2	1.43726	1	121.32962		
N	2	2.58848	1	121.57853	3	359.46953
N	1	1.24229	2	69.87321	3	258.90867
H	2	1.08195	1	120.50129	3	200.07763
H	1	1.08005	2	149.20540	3	77.94024

^1c-$CH(N)CHCN$

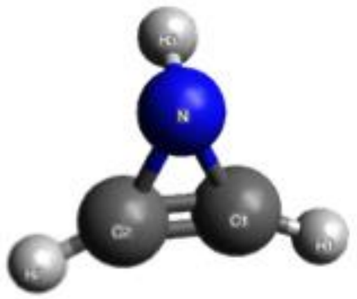

C						
C	1	1.26892				
N	2	1.51635	1	65.26729		
H	1	1.07113	2	156.29394	3	168.99135
H	2	1.07114	1	156.29124	3	190.94153
H	3	1.02345	2	107.78894	1	98.43148

^1c-CH(NH)CH

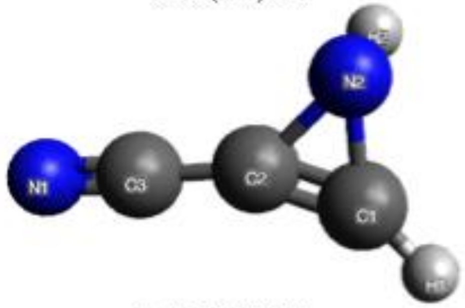

C						
C	1	1.27748				
C	2	1.38034	1	158.14962		
N	2	2.53595	1	158.77554	3	359.07492
N	1	1.47473	2	68.00948	3	189.22702
H	1	1.07388	2	153.37110	3	3.00824
H	5	1.02396	1	108.46518	2	261.73341

^1c-CH(NH)CCN

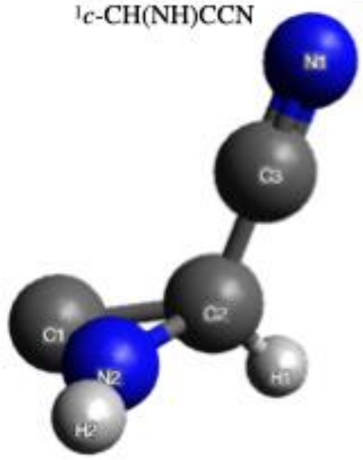

C						
C	1	1.61315				
C	2	1.43329	1	119.71668		
N	2	2.58520	1	119.97382	3	359.69485
N	1	1.27313	2	57.84089	3	255.59851
H	2	1.08077	1	118.07308	3	208.40222
H	5	1.01231	1	144.15590	2	176.88069

^1c-C(NH)CHCN

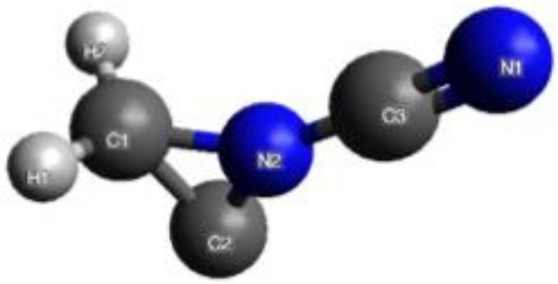

C						
C	1	1.55299				
C	2	2.52120	1	76.93947		
N	3	1.15348	2	165.28649	1	179.99707
N	2	1.30617	1	59.87601	3	359.99718
H	1	1.08252	2	118.84789	3	257.39776
H	1	1.08249	2	118.88616	3	102.60235

^1c-CH_2(NCN)C

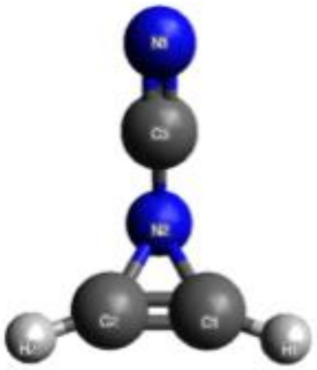

C						
C	1	1.26626				
C	2	2.42896	1	74.91384		
N	3	1.15538	2	150.06958	1	241.95124
N	3	1.35964	2	34.81413	1	67.13365
H	1	1.07119	2	158.90927	3	154.05621
H	2	1.07121	1	158.87595	3	205.85137

[1]*c*-CH(NCN)CH

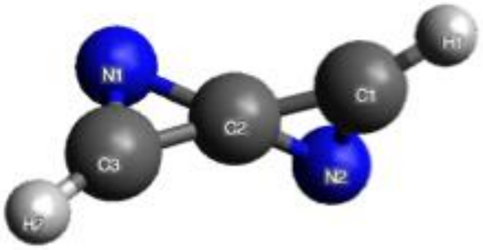

C						
C	1	1.40977				
C	2	1.40973	1	156.41573		
N	3	1.27159	2	67.02909	1	133.10070
N	1	1.27167	2	67.03154	3	133.31860
H	1	1.08360	2	155.75104	3	311.63757
H	3	1.08355	2	155.64791	1	311.14521

[1]*c*-CH(N)CCHN

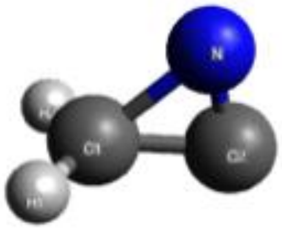

C						
C	1	1.49406				
N	2	1.21141	1	73.94261		
H	1	1.07956	2	119.98477	3	263.58621
H	1	1.07956	2	119.98477	3	96.41379

c-CH_2(N)C

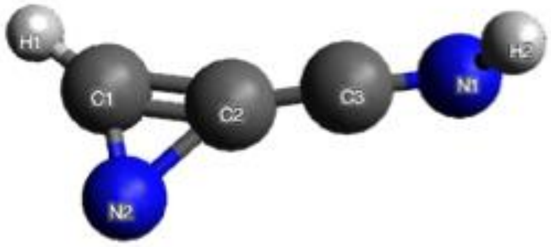

C						
C	1	1.39593				
C	2	1.27926	1	164.53064		
N	3	1.23446	2	171.39859	1	257.31372
N	1	1.25832	2	70.47377	3	193.78538
H	1	1.08162	2	149.91244	3	14.14489
H	4	1.02061	3	114.62383	2	179.35090

[1]*c*-CH(N)CCNH

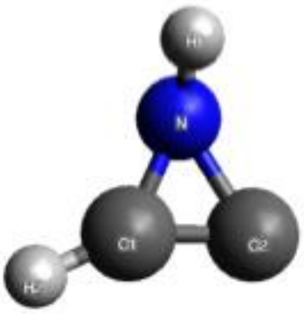

c-CH(NH)C

C						
C	1	1.35584				
N	1	1.36149	2	66.65354		
H	3	1.01774	1	130.23113	2	247.92021
H	1	1.07428	2	155.01873	3	182.68779

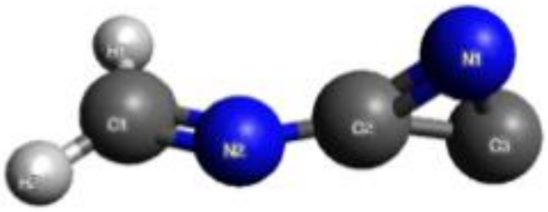

[1]*c*-$CH_2NC(N)C$

C						
C	1	2.43211				
C	2	1.44644	1	157.18401		
N	3	1.32526	2	57.84292	1	153.58133
N	2	1.25621	1	15.31859	3	241.97085
H	1	1.08626	2	106.38601	3	295.23277
H	1	1.08292	2	134.42274	3	114.28664

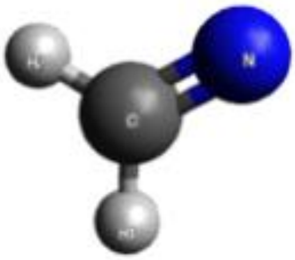

H_2CN

C						
N	1	1.23794				
H	1	1.09755	2	121.50867		
H	1	1.09731	2	121.56479	3	179.99825

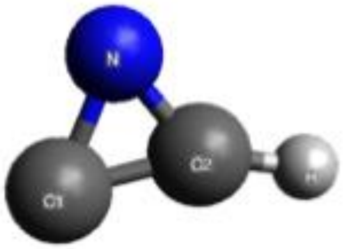

[1]*c*-C(N)CH

C						
C	1	1.39644				
N	2	1.29057	1	62.81549		
H	2	1.07879	1	158.97676	3	179.98521

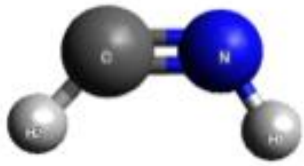

HCNH

C						
N	1	1.21760				
H	2	1.02522	1	122.72699		
H	1	1.09688	2	133.98864	3	0.00000

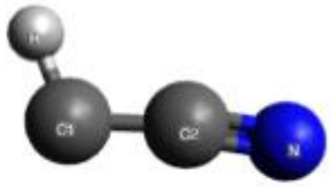

^{1}HCCN

C						
C	1	1.36792				
N	2	1.17415	1	172.29129		
H	1	1.09503	2	112.11024	3	180.02822

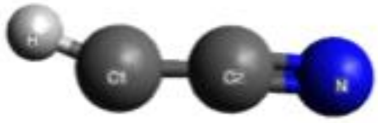

^{3}HCCN

C						
C	1	1.29425				
N	2	1.19838	1	176.18673		
H	1	1.06573	2	157.71586	3	180.03147

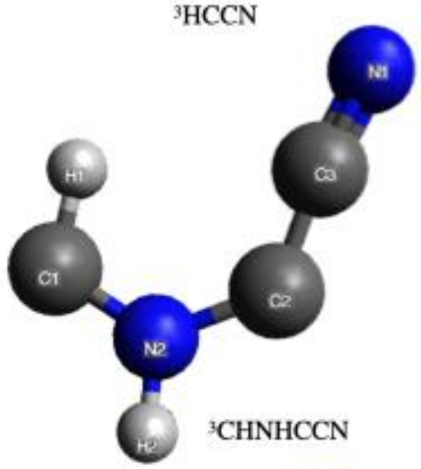

^{3}CHNHCCN

C						
C	1	2.39168				
C	2	1.35405	1	103.99304		
N	3	1.17287	2	172.46910	1	180.02512
N	1	1.31772	2	28.03981	3	179.97611
H	1	1.09683	2	83.46943	3	359.96548
H	5	1.01182	1	118.79414	2	180.00277

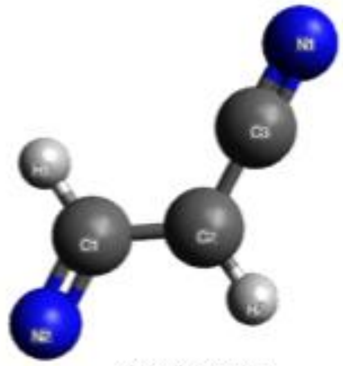

^{3}CHNCHCN

C						
C	1	1.41820				
C	2	1.39589	1	122.13182		
N	2	2.55565	1	122.26147	3	0.00002
N	1	1.27850	2	123.08707	3	179.99999
H	1	1.09487	2	118.56283	3	0.00007
H	2	1.08128	1	119.44159	3	179.99993

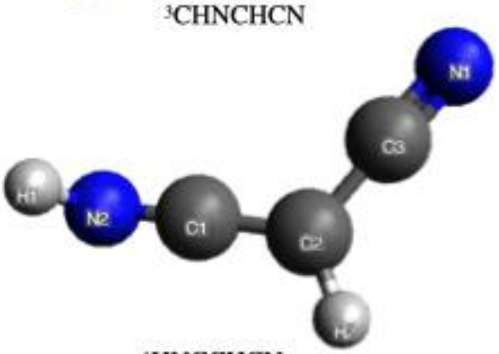

^{1}HNCCHCN

C						
C	1	1.32092				
C	2	1.41538	1	122.18906		
N	2	2.56874	1	122.92746	3	0.07214
N	1	1.20617	2	173.73335	3	99.66556
H	5	1.01642	1	120.18978	2	171.50080
H	2	1.08196	1	118.71690	3	178.82558

C						
C	1	1.41846				
C	2	1.39911	1	123.69764		
N	2	2.55704	1	124.56582	3	359.99993
N	1	1.27901	2	125.50992	3	359.99993
H	1	1.09499	2	116.86370	3	179.99993
H	2	1.08114	1	119.04772	3	180.00007

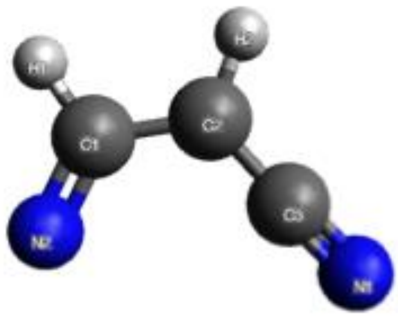

^{3}NCHCHCN

C						
C	1	2.33501				
C	2	1.34561	1	124.26384		
N	3	1.17442	2	172.23196	1	180.00085
N	2	1.26859	1	25.55225	3	180.00011
H	1	1.08091	2	143.19965	3	180.00213
H	1	1.08824	2	97.00451	3	359.99857

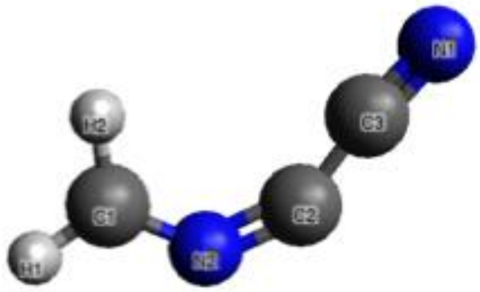

$^{3}CH_2NCCN$

C						
C	1	2.47862				
C	2	1.39367	1	132.21286		
N	3	1.15795	2	172.71374	1	180.03762
N	3	2.35472	1	7.20773	2	359.94081
H	1	1.08134	2	119.50762	3	268.46003
H	1	1.08141	2	119.47863	3	91.58547

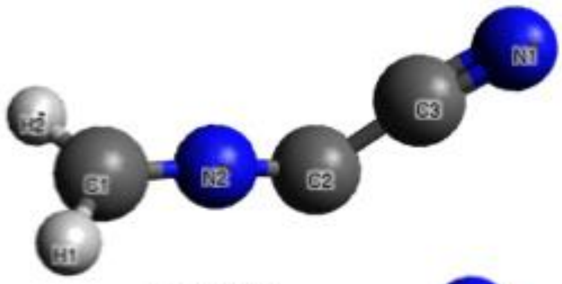

$^{1}CH_2NCCN$

C						
C	1	2.48305				
C	2	1.41159	1	121.21878		
N	2	2.56648	1	122.04340	3	0.00305
N	3	2.35551	1	10.43332	2	349.07557
H	1	1.08454	2	129.91575	3	267.99654
H	2	1.07988	1	118.46282	3	183.94401

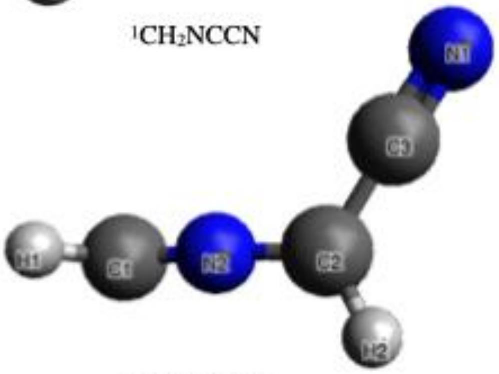

^{1}CHNCHCN

C						
C	1	1.31353				
N	2	1.23870	1	174.05770		
H	3	1.02583	2	113.96205	1	180.49088

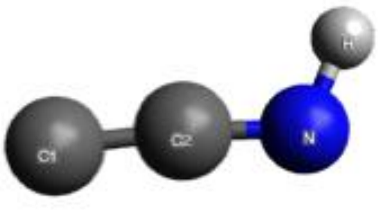

^{1}CCNH

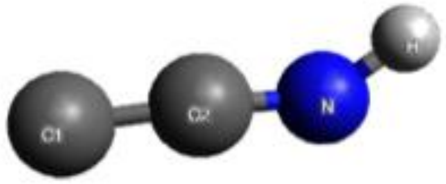

C						
C	1	1.33491				
N	2	1.20865	1	174.54877		
H	3	1.00571	2	137.79861	1	179.99498

^{3}CCNH

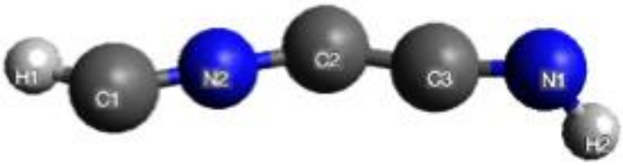

^{1}CHNCCNH

C						
C	1	2.44359				
C	2	1.27076	1	151.67337		
N	3	1.22768	2	171.58546	1	234.70230
N	3	2.45637	2	13.78431	1	0.58631
H	1	1.07240	2	141.40903	3	186.20970
H	4	1.01336	3	119.71047	2	209.90372

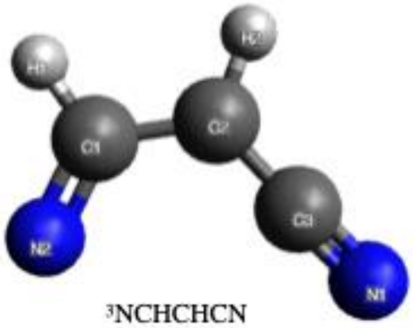

C						
C	1	1.41846				
C	2	1.39911	1	123.69764		
N	2	2.55704	1	124.56582	3	359.99993
N	1	1.27901	2	125.50992	3	359.99993
H	1	1.09499	2	116.86370	3	179.99993
H	2	1.08114	1	119.04772	3	180.00007

^{3}NCHCHCN

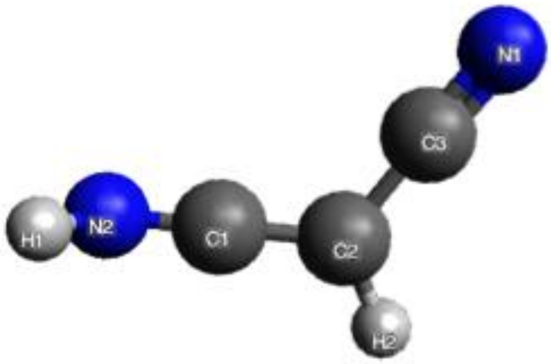

C						
C	1	1.32092				
C	2	1.41538	1	122.18906		
N	2	2.56874	1	122.92746	3	0.07214
N	1	1.20617	2	173.73335	3	99.66556
H	5	1.01642	1	120.18978	2	171.50080
H	2	1.08196	1	118.71690	3	178.82558

^{1}NHCCHCN

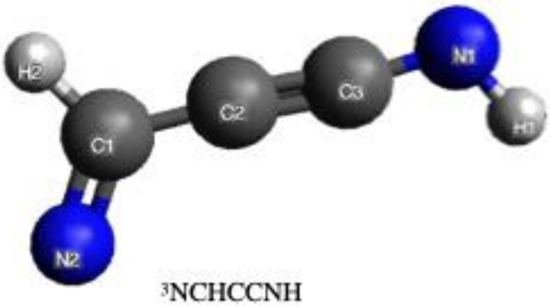

C						
C	1	1.39345				
C	2	1.25020	1	175.61171		
N	3	1.24956	2	172.71029	1	179.24532
N	1	1.27951	2	122.64380	3	0.81057
H	4	1.01910	3	114.78123	2	179.99321
H	1	1.09687	2	119.49238	3	180.69071

^{3}NCHCCNH

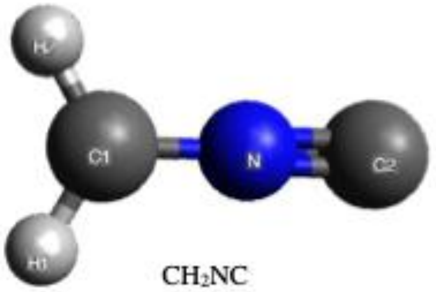

CH_2NC

C						
C	1	2.51428				
N	2	1.18182	1	0.09347		
H	1	1.07856	2	118.77897	3	179.69967
H	1	1.07856	2	119.12869	3	359.69884

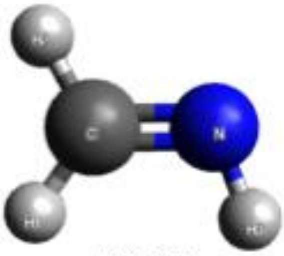

$^1CH_2NH$

C						
N	1	1.26390				
H	1	1.09345	2	124.67646		
H	1	1.08904	2	118.96024	3	179.97395
H	2	1.02046	1	111.40702	3	359.97675

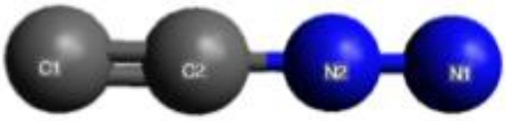

CCNN

C						
C	1	1.26282				
N	2	2.38880	1	179.97438		
N	3	1.12287	2	0.02562	1	0.00000

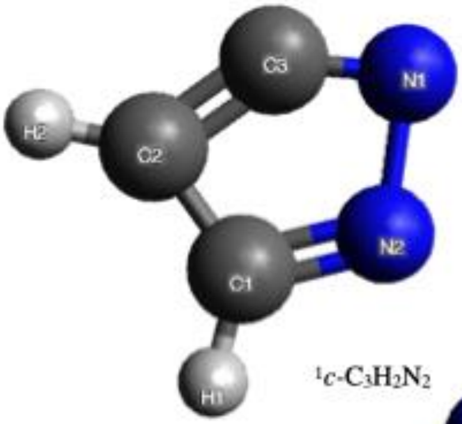

^1c-$C_3H_2N_2$

C						
C	1	1.39497				
H	1	1.07908	2	130.25993		
C	2	1.37079	1	90.18834	3	179.98758
H	2	1.06974	1	136.97624	3	0.00634
N	4	1.23482	2	138.09125	1	0.00726
N	1	1.36238	2	111.73532	3	179.99314

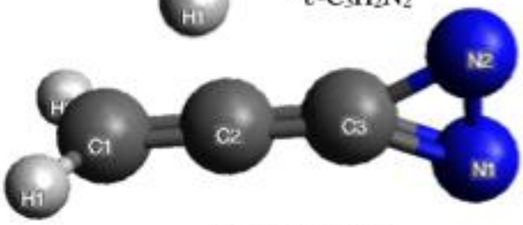

^1c-$CH_2CC(N)N$

C						
C	1	1.30671				
H	1	1.08359	2	121.45368		
H	1	1.08367	2	121.44907	3	179.92772
C	3	3.27726	1	42.17991	2	359.95324
N	5	1.42140	2	154.04608	1	26.82282
N	6	1.24275	5	64.08693	2	180.07446

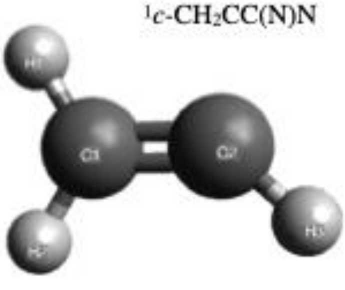

CH_2CH

C						
C	1	1.30127				
H	1	1.08577	2	122.28669		
H	1	1.09129	2	121.83822	3	180.06320
H	2	1.07749	1	138.78445	3	180.08060
